# KDM5 histone demethylases repress immune response via suppression of STING

**DOI:** 10.1371/journal.pbio.2006134

**Published:** 2018-08-06

**Authors:** Lizhen Wu, Jian Cao, Wesley L. Cai, Sabine M. Lang, John R. Horton, Daniel J. Jansen, Zongzhi Z. Liu, Jocelyn F. Chen, Meiling Zhang, Bryan T. Mott, Katherine Pohida, Ganesha Rai, Stephen C. Kales, Mark J. Henderson, Xin Hu, Ajit Jadhav, David J. Maloney, Anton Simeonov, Shu Zhu, Akiko Iwasaki, Matthew D. Hall, Xiaodong Cheng, Gerald S. Shadel, Qin Yan

**Affiliations:** 1 Department of Pathology, Yale School of Medicine, New Haven, Connecticut, United States of America; 2 Department of Molecular and Cellular Oncology, The University of Texas MD Anderson Cancer Center, Houston, Texas, United States of America; 3 National Center for Advancing Translational Sciences, National Institutes of Health, Rockville, Maryland, United States of America; 4 Institute of Immunology and the CAS Key Laboratory of Innate Immunity and Chronic Disease, School of Life Sciences and Medical Center, University of Science and Technology of China, Hefei, China; 5 Department of Immunobiology, Yale School of Medicine, New Haven, Connecticut, United States of America; 6 Howard Hughes Medical Institute, Chevy Chase, Maryland, United States of America; 7 Department of Genetics, Yale School of Medicine, New Haven, Connecticut, United States of America; 8 Salk Institute for Biological Studies, La Jolla, California, United States of America; New York University, United States of America

## Abstract

Cyclic GMP-AMP (cGAMP) synthase (cGAS) stimulator of interferon genes (STING) senses pathogen-derived or abnormal self-DNA in the cytosol and triggers an innate immune defense against microbial infection and cancer. STING agonists induce both innate and adaptive immune responses and are a new class of cancer immunotherapy agents tested in multiple clinical trials. However, *STING* is commonly silenced in cancer cells via unclear mechanisms, limiting the application of these agonists. Here, we report that the expression of *STING* is epigenetically suppressed by the histone H3K4 lysine demethylases KDM5B and KDM5C and is activated by the opposing H3K4 methyltransferases. The induction of *STING* expression by KDM5 blockade triggered a robust interferon response in a cytosolic DNA-dependent manner in breast cancer cells. This response resulted in resistance to infection by DNA and RNA viruses. In human tumors, *KDM5B* expression is inversely associated with *STING* expression in multiple cancer types, with the level of intratumoral CD8^+^ T cells, and with patient survival in cancers with a high level of cytosolic DNA, such as human papilloma virus (HPV)-positive head and neck cancer. These results demonstrate a novel epigenetic regulatory pathway of immune response and suggest that KDM5 demethylases are potential targets for antipathogen treatment and anticancer immunotherapy.

## Author summary

Pathogens often find ways to turn down cell-intrinsic antipathogen immune responses by the host. Similarly, cancer cells use various mechanisms to evade attack by immune cells. One of the common mechanisms is suppression of the stimulator of interferon genes (STING)-dependent innate immune response. Using potent and specific small-molecule inhibitors and genetic-depletion approaches, we found that the silenced STING pathway can be reactivated in breast cancer cells by suppressing KDM5 demethylases. Activation of the STING pathway led to a robust interferon response, which blocked viral infection, and was associated with increased tumor-infiltrated lymphocytes and better patient survival in multiple cancer types. This discovery has major clinical implications for treating both pathogen infection and cancer because KDM5 inhibition provides a fast, robust, and reversible control of innate immune response. Since the discovery of histone demethylase activity of KDM5 proteins a decade ago, significant efforts have been dedicated to developing KDM5 inhibitors for clinical applications. In fact, a KDM5 inhibitor recently entered phase I clinical trial for treatment of hepatitis B infection. Here, we provide mechanistic insights on how KDM5 inhibitors block viral infection. Moreover, our results suggest that KDM5 inhibitors can also be combined with other cancer immunotherapies.

## Introduction

Evasion from immunosurveillance by cancer cells is a major cancer hallmark [1], and restoration of immunosurveillance has been demonstrated as an effective antitumor strategy. For example, antibodies targeting inhibitory checkpoint molecules, including programmed cell death protein 1 (PD-1) and cytotoxic T-cell lymphocyte-associated protein 4 (CTLA-4), have achieved remarkable efficacy in the clinic [2]. However, only a small percentage of patients respond to these therapies. Thus, the mechanisms for lack of response to these treatments are areas of intense investigation. Lack of T-cell infiltration (also known as immunologically “cold” tumors) appears to characterize a major subset of patients who do not respond to treatment [3]. Identification of strategies that convert tumors from an immunologically “cold” to “hot” state could enhance immune checkpoint inhibitor therapies and potentially result in the effective treatment of patients who otherwise would not have responded.

Pattern recognition receptors (PRR) are cell surface and intracellular sensors that recognize pathogen-associated and abnormal-self molecular patterns, e.g., nucleic acids, and trigger intracellular signaling cascades to activate cell-intrinsic antipathogen or antitumor responses [4]. Cyclic GMP-AMP (cGAMP) synthase (cGAS) senses pathogen- or abnormally released self-DNA [5, 6] and signals through stimulator of interferon genes (STING) [7]. RNA helicases retinoic acid inducible gene I (RIG-I) and melanoma differentiation-associated gene 5 (MDA5) are the main cytosolic RNA sensors—and activate the interferon pathway through mitochondrial antiviral signaling protein (MAVS)—whereas toll-like receptors (TLRs) respond to pathogen-associated molecular patterns on the cell surface or in endosomal compartments [4]. The downstream pathway of these diverse receptors converges on a few key transcription factors called interferon regulatory factors (notably IRF3 and IRF7) and protein kinases (such as TANK-binding kinase 1 [TBK1]) responsible for the phosphorylation and nuclear translocation of IRF3 and IRF7 [8]. Activated IRFs drive the transcription of type I interferons, which bind to their cognate cell surface receptors and lead to the formation of the canonical signal transducer and activator of transcription 1 (STAT1)–STAT2–IRF9 (also known as interferon-stimulated gene factor 3 [ISGF3]) complex. The ISGF3 complex binds to the promoters of interferon-stimulated genes (ISGs) and activates these genes, many of which mediate the immune response [8]. Emerging evidence suggests that the cGAS-STING pathway plays a critical role in bridging innate immunity and adaptive immunity in tumors [9–11]. However, this pathway is silenced in many tumors, and the mechanisms of their silencing remain largely unknown [12–15].

Tri-methylation on histone H3 lysine 4 (H3K4me3) is enriched near transcription start sites and strongly correlates with active transcription [16]. Methylation on H3K4, like other histone marks, is dynamically controlled through the concerted action of lysine methyltransferases, the writers, and demethylases, the erasers [16]. The lysine demethylase 5 (KDM5) family proteins—including KDM5A-D (also known as JARID1A-D)—are Fe (II)- and α-ketoglutarate-dependent dioxygenases and catalyze the removal of the methyl groups from H3K4me3 [17]. The KDM5 family demethylases play major roles in human cancers. KDM5A physically and functionally interacts with tumor suppressor pRb [18]. *KDM5B* is up-regulated in breast cancer cells overexpressing the *ERBB2/HER2* oncogene [19]. Gene amplification of both *KDM5A* and *KDM5B* were found in various human cancers [20, 21]. Studies using cancer cell lines and mouse models demonstrated their functions in promoting tumorigenesis in multiple cancer types [17, 21–29]. However, the mechanisms by which KDM5 proteins contribute to these phenotypes are still largely unclear.

Here, we report that KDM5 demethylases suppress STING-induced innate immune response in tumor cells. We found that KDM5B and KDM5C bind to the *STING* locus and maintains a low level of H3K4me3 to suppress *STING* expression. Inhibition or depletion of KDM5B and KDM5C led to increased *STING* expression in a wide range of cancer cells. In the presence of abnormal cytosolic DNA, the increased STING led to a robust induction of ISGs in breast cancer cells and antiviral response through the cGAS-STING-TBK1-IRF3 pathway. Lastly, we found a strong negative correlation between *KDM5B* expression and *STING* expression in The Cancer Genome Atlas (TCGA) tumor samples. Our findings reveal a novel epigenetic suppressive mechanism of innate immune response and suggest KDM5 demethylases as attractive targets to boost antitumor immune response.

## Results

### KDM5 inhibition or depletion activates ISGs

**Fig 1:**
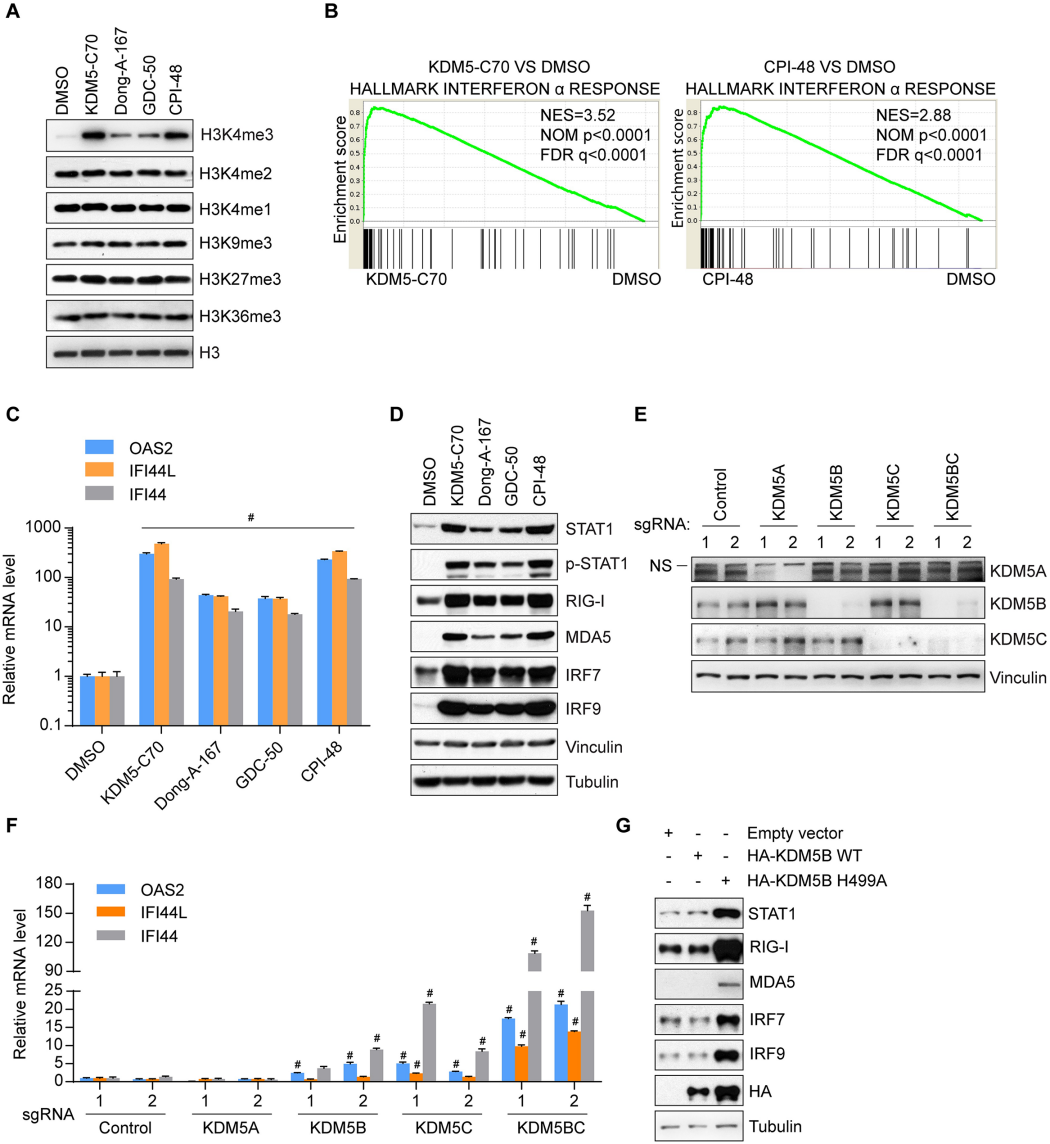
Inhibition of KDM5 demethylases activates interferon-induced genes. (A) Western blot analysis of histone modifications in MCF7 cells treated with 1 μM KDM5-C70, 10 μM Dong-A-167, 10 μM GDC-50, or 10 μM CPI-48 for 3 days. (B) GSEA of RNA-seq data from MCF7 cells treated with 3 μM KDM5-C70 or CPI-48 for 6 days. (C, D) RT-qPCR (panel C) and western blot (panel D) analyses of MCF7 cells treated with 1 μM KDM5-C70, 10 μM Dong-A-167, 10 μM GDC-50, or 10 μM CPI-48 for 6 days. (E, F) Western blot (panel E) and RT-qPCR (panel F) analyses of MCF7 cells with stable knockout of the indicated genes using the CRISPR/Cas9 system. Control 1, empty vector; control 2, scrambled sequence. KDM5BC, 2 sgRNAs targeting KDM5B and KDM5C. (G) Western blot analysis of MCF7 cells after transfection with indicated plasmids. Representative data from triplicate experiments are shown. Error bar denotes SEM. ^#^*p* < 0.01 for inhibitors versus DMSO (panel C), and for knockout sgRNA versus average of 2 control sgRNAs (panel F). The numerical values used to generate graphs in panel C and F are available in S1 Data. CRSPR/Cas9, clustered regular interspaced short palindromic repeats/CRISPR-associated protein 9; FDR q, false discovery rate q value; GSEA, gene set enrichment analysis; NES, normalized enrichment score; NOM p, nominal p value; NS, nonspecific band; RNA-seq, RNA sequencing; RT-qPCR, reverse transcription followed by quantitative PCR; sgRNA, single guide RNA.

All 4 family members of KDM5 demethylases (KDM5A-D) share sequence and structure similarity [17], have similar in vitro kinetic parameters [30], and display functional redundancy [31]. Depletion of individual KDM5 enzymes usually alters histone modification level and gene expression in a context-dependent manner [17], but the effects of inhibiting multiple KDM5 enzymes remain unclear. Multiple potent pan-KDM5 inhibitors—including KDM5-C49 (cell active form is KDM5-C70) [30, 32], Dong-A-167 (patent WO2016068580), GDC-50 [33], and CPI-48 [34]—have been reported. These inhibitors are known or predicted to compete with the cofactor α-ketoglutarate in the active site of KDM5 enzymes (S1A–S1F Fig and S1 Table) and inhibited KDM5 enzymes with half maximal inhibitory concentration (IC_50_) values in the nM range (S1G and S1H Fig and S1 Data). We examined the effects of these small-molecule inhibitors on histone modifications and gene expression in MCF7 breast cancer cells. First, global levels of H3K4me3 increased in inhibitor-treated cells (Fig 1A), consistent with previous results [30, 32–36]. Second, these inhibitors showed minimal effects on other histone methylation marks, including tri-methylation on histone H3 lysine 9 (H3K9me3—a substrate for the KDM4 family), lysine 27 (H3K27me3—a substrate for the KDM6 family), and lysine 36 (H3K36me3—another substrate for the KDM4 family), as well as di- or mono-methylation on histone H3 lysine 4 (H3K4me2/me1, substrates for the KDM1/LSD and KDM5 family) (Fig 1A). Third, KDM5-C70 treatment induced KDM5B and KDM5C protein levels without affecting KDM5A protein level (S2A Fig). It is possible that the induction of KDM5B and KDM5C is due to a feedback regulation, and the mechanism of their differential induction will require further investigation. Fourth, despite the global increase of H3K4me3, RNA sequencing (RNA-seq) analysis of MCF7 cells treated with inhibitors KDM5-C70 and CPI-48 revealed major up-regulation of gene expression only in limited pathways (S2B Fig). The top up-regulated genes are involved in the interferon response pathway (Fig 1B, S2B Fig and S2 and S3 Data). Reverse transcription followed by quantitative PCR (RT-qPCR) analysis detected a robust increase of ISGs with direct antiviral activities, such as *OAS2*, *IFI44L*, *IFI44*, *IFIT1*, and *IFIT3*, and chemokine genes involved in immune cell recruitment, such as *CXCL10*, upon treatment with inhibitors (Fig 1C and S2C Fig).

Phosphorylated STAT1, which is often required for induction of ISGs [8], increased along with total STAT1 (Fig 1D). Consistently, other genes involved in type I interferon response were up-regulated, including cytosolic RNA sensors RIG-I and MDA5, and interferon-regulatory factors IRF7 and IRF9 (Fig 1D and S2C Fig). Treatment of other breast cancer cells SKBR3 and BT474 by compound KDM5-C70 also induced expression of *OAS2*, *IFI44L*, and *IFI44*, but to a lesser extent (S2D and S2E Fig). We noted that compound KDM5-C70 at 1 μM significantly induced a global change of H3K4me3 level and targeted gene expression, whereas the other 3 compounds at 10 μM showed similar (or less) potency (Fig 1A and 1D), therefore we used 1 μM KDM5-C70 in the remaining study.

Depletion of KDM5B or KDM5C, but not KDM5A, mediated by clustered regular interspaced short palindromic repeats/CRISPR-associated protein 9 (CRISPR/Cas9) led to moderately increased expression of ISGs, and knockout of *KDM5B* and *KDM5C* synergistically enhanced their expression (Fig 1E and 1F). *KDM5D* is located in the Y chromosome [17] and thus not expressed in breast cancer cells derived from female patients. Similar effects were observed in cells with small interfering RNA (siRNA)-mediated individual and combinatorial knockdown of *KDM5B* and *KDM5C* demethylases (S2F and S2G Fig). Compared to the effects of KDM5 inhibitor treatment, the magnitude of ISG activation was slightly lower in *KDM5B* and *KDM5C* double knockout cells. It may be due to incomplete depletion of KDM5B and KDM5C in polyclonal knockout cells that we used. Activation of negative feedback pathways during the time required to generate stable cell lines could have also dampened the effects.

Ectopic overexpression of a catalytic deficient KDM5B mutant (H499A), but not wild-type KDM5B, dramatically activated expression of ISGs (Fig 1G), suggesting that this KDM5B mutant had dominant negative effects. Collectively, these results showed that the demethylase activities of KDM5B and KDM5C are required to inhibit the interferon pathway.

### Loss of KDM5 demethylases primes the antiviral innate immune response

**Fig 2:**
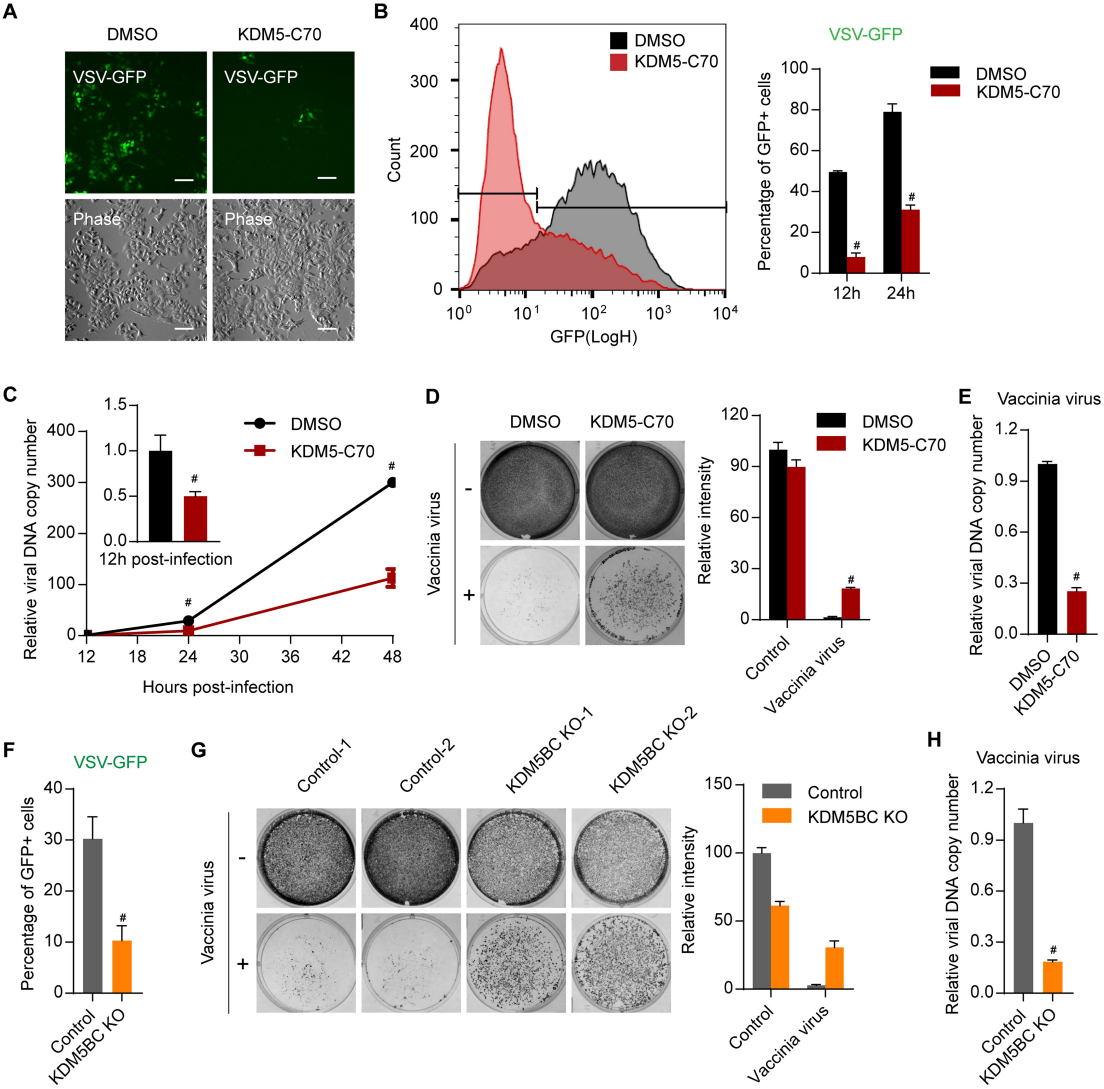
Inhibition of KDM5 demethylases primes the innate antiviral immune response. (A) Representative images of MCF7 cells 24 hours after infection with VSV-GFP viruses at MOI 0.5. Scale bar, 100 μm. (B) Flow cytometry plot (left panel, 24 hours) and quantification of GFP-positive cells (right panel) after infection with VSV-GFP virus for the indicated time at MOI 0.5. (C) qPCR analysis of DNA copy number of vaccinia virus in MCF7 cells at the indicated time after infection at MOI 0.25. (D) Representative crystal violet staining images (left panel) and quantification of relative intensity (right panel) of MCF7 cells 3 days after infection with vaccinia virus at MOI 0.5. (E) qPCR analysis of DNA copy number of vaccinia virus in growth media from the cells shown in panel D. (F) Quantification of flow cytometry analysis for the percentage of GFP-positive cells in control or KDM5B and KDM5C double knockout cells 12 hours after infection with VSV-GFP virus at MOI 0.5. (G) Representative crystal violet staining images (left panel) and quantification of relative intensity (right panel) of the indicated MCF7 knockout cells 3 days after infection with vaccinia virus at MOI 0.25. (H) qPCR analysis of DNA copy number of vaccinia virus in growth media from the cells shown in panel G. Cells were pretreated with DMSO or 1 μM KDM5-C70 for 5 days, followed by no treatment for 1 day, before viral infection in panel A–E. Representative data from triplicate experiments are shown in panel C, E, F, and H. Two or 3 biological replicates are shown in panel B, D, and G. Error bar denotes SEM. ^#^*p* < 0.01. The numerical values used to generate graphs in B–H are available in S1 Data. MOI, multiplicity of infection; qPCR, quantitative PCR; VSV-GFP, vesicular stomatitis virus carrying a green fluorescent protein reporter.

It is well-known that type I interferon establishes an antiviral state [8]. To assess the biological outcome of interferon response induced by KDM5 inhibition, we challenged inhibitor-treated cells with vesicular stomatitis virus (VSV, a negative-stranded RNA virus) carrying a green fluorescence protein (GFP) reporter (VSV-GFP) or vaccinia virus (a double-stranded DNA [dsDNA] virus). Infection by both viruses can be suppressed by treatment with type I interferons [37, 38]. To exclude the direct effects of KDM5 inhibition on viral infection or reproduction, KDM5-C70 was removed 1 day before infection. We found that pretreatment of cells with KDM5-C70 significantly inhibited VSV-GFP infection (Fig 2A and 2B). Similarly, analyzing the copy number of the viral genome at different time points after vaccinia virus infection revealed that viral replication was significantly restrained in inhibitor-pretreated cells (Fig 2C). As a result, inhibitor-pretreated cells resisted some lytic effects of vaccinia virus (Fig 2D) and produced much fewer viruses compared with control cells (Fig 2E). Similar results were obtained when KDM5B and KDM5C were depleted by CRIPSR/Cas9-mediated knockout (Fig 2F–2H). In summary, inhibition of KDM5 enzymes potentiates antiviral innate immunity.

### Activation of ISGs by KDM5 inhibition is dependent on the cGAS-STING-TBK1-IRF3 signaling cascade

**Fig 3:**
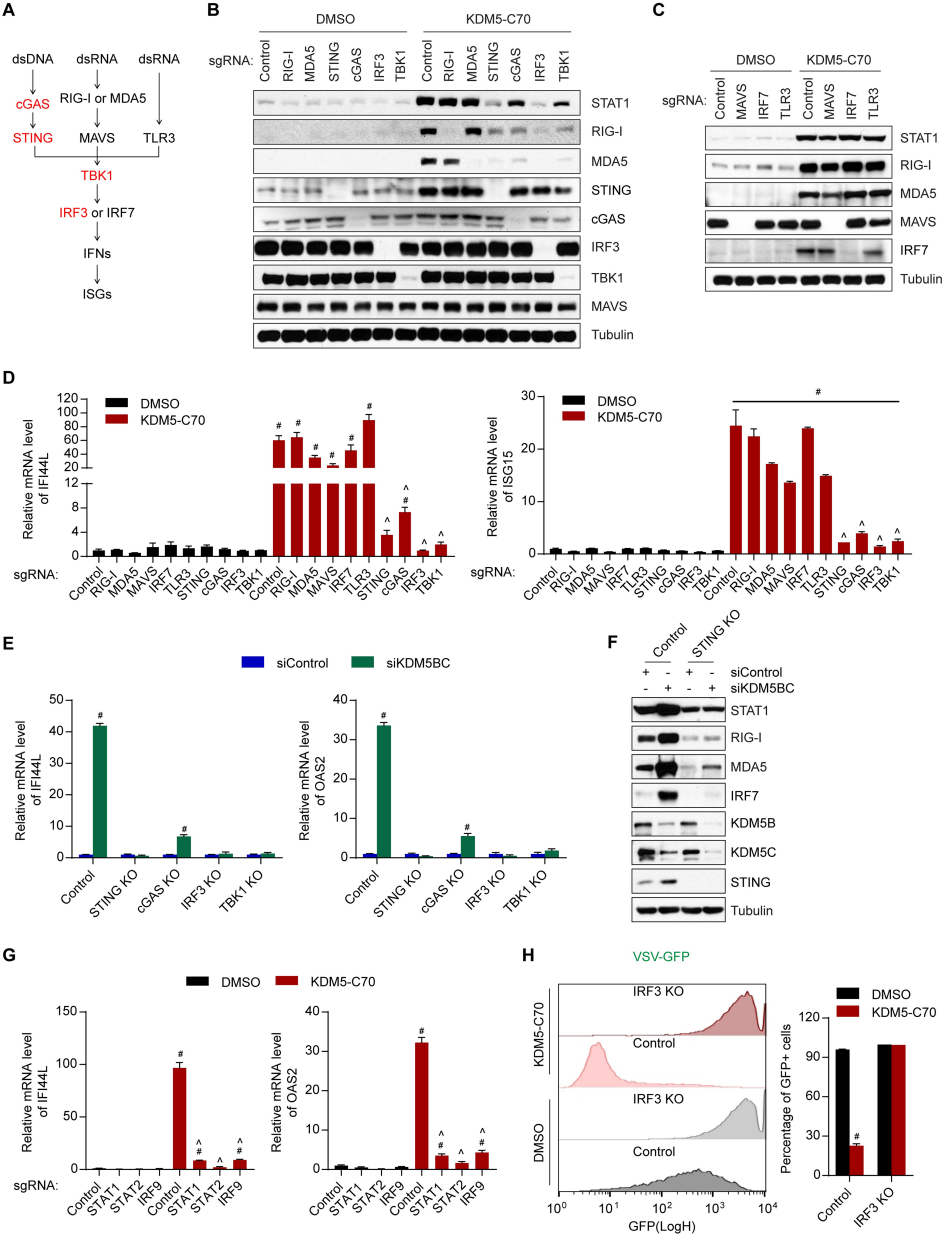
Activation of ISGs by KDM5 inhibition is dependent on the cGAS-STING-TBK1-IRF3 signaling pathway. (A) Schematic of the pattern recognition receptor pathways. (B–D) Western blot (panel B and C) and RT-qPCR (panel D) analyses of MCF7 cells with knockout of the indicated genes after treatment with DMSO or 1 μM KDM5-C70 for 6 days. (E, F) RT-qPCR (panel E) and western blot (panel F) analyses of MCF7 cells with knockout of the indicated genes 5 days after transfection with the indicated siRNAs. (G) RT-qPCR analysis of MCF7 cells with knockout of the indicated genes after treatment with DMSO or 1 μM KDM5-C70 for 6 days. (H) Flow cytometry plots (left panel) and quantification of percentage of GFP-positive cells (right panel) in the indicated MCF7 knockout cells 24 hours after infection with VSV-GFP virus at MOI 0.5. Error bar denotes SEM. Representative data from triplicate experiments are shown in panel D, E, and G. Three biological replicates are shown in panel H. ^#^*p* < 0.01 for inhibitors versus DMSO (panel D, G, and H), knockdown of KDM5B and KDM5C versus control (panel E). ^*p* < 0.01 for knockout sgRNA versus control sgRNA (panel D and G). The numerical values used to generate graphs in panel D, E, G, and H are available in S1 Data. cGAS, cyclic GMP-AMP synthase; IRF3, interferon regulatory factor 3; ISG, interferon-stimulated gene; MOI, multiplicity of infection; RT-qPCR, reverse transcription followed by quantitative PCR; sgRNA, single guide RNA; siRNA, small interfering RNA; STING, stimulator of interferon genes; TBK1, TANK-binding kinase 1; VSV-GFP, vesicular stomatitis virus carrying a green fluorescent protein reporter.

We next examined which pathway is required for the interferon response triggered by KDM5 inhibition. Using the CRISPR/Cas9 system, we depleted major components in the interferon-inducing PRR pathways individually, including RIG-I, MDA5, MAVS, TBK1, IRF3, IRF7, cGAS, STING, and TLR3 (Fig 3A–3C). Efficient knockout of these genes was achieved in polyclonal setting as shown by western blot (Fig 3B and 3C) or T7 endonuclease assay (S3A Fig). Depletion of cGAS, STING, IRF3, or TBK1 largely abolished KDM5-C70-induced expression of *IFI44L*, *ISG15*, and other ISGs (Fig 3B–3D and S3B and S3C Fig). In contrast, loss of RIG-I, MDA5, MAVS, IRF7, or TLR3 had minimal effect (Fig 3B–3D). We note that some components in these pathways—including RIG-I, MDA5, and IRF7—are ISG products themselves, and KDM5-C70 treatment induced the expression of these proteins as well (Figs 1D, 3B and 3C and S2C Fig). Consistently, knockout of essential components in the KDM5-C70-triggered interferon response—such as *IRF3* and *TBK1*—blocked the induction of RIG-I and MDA5 (Fig 3B). These data highlight the predominant roles of cGAS-STING in KDM5-inhibition–dependent activation of ISGs. To further confirm the requirement of the cGAS-STING-TBK1-IRF3 signaling pathway for the KDM5-inhibitor–induced interferon response, we conducted combinatorial knockdown of *KDM5B* and *KDM5C* in *cGAS*, *STING*, *TBK1*, or *IRF3* knockout cells. Loss of any of the components in this signaling pathway was sufficient to blunt the KDM5B/C-loss–induced interferon response (Fig 3E and 3F and S3D Fig). Together, these data suggest that activation of interferon response by KDM5 deficiency is dependent on the cGAS-STING-TBK1-IRF3 signaling cascade rather than on direct modulation of ISG expression.

Consistent with the activation of ISGs in KDM5 inhibitor-treated cells, we observed increased expression of type I interferon and IFN-β, as well as type III interferons IFN-λ1 and IFN-λ2, in response to KDM5-C70 treatment (S3E Fig). We also compared the effects of KDM5-C70 treatment to 5 to 500 unit/ml IFN-β treatment on the expression levels of 32 ISGs, most of which have antiviral activity [39]. We found that KDM5 inhibition induced similar patterns of ISGs as IFN-β treatment, and the extent of ISG induction upon KDM5 inhibition is similar to 25 unit/ml IFN-β treatment (S3F Fig). Knockout of individual components of the cGAS-STING-TBK1-IRF3 signaling pathway significantly blocked the effect of KDM5-C70 on the induction of interferons (S3E Fig). Moreover, conditioned media collected from inhibitor-pretreated control MCF7 cells—but not from cGAS-, STING-, TBK1-, or IRF3-deficient cells—were able to activate ISG expression in inhibitor-untreated cells (S3G Fig). Furthermore, loss of any member of the ISGF3 complex, namely STAT1, STAT2, and IRF9, blocked the effects of KDM5-C70 (Fig 3G and S3H Fig). Taken together, our data suggest that inhibition of KDM5 enzymes facilitates the cGAS-STING-TBK1-IRF3 signaling cascade to trigger an interferon response, resulting in increased secretion of interferons and activation of the ISGF3 complex to induce the expression of ISGs.

To further determine whether the resistance to viral infection by KDM5 inhibition was also dependent on cGAS-STING-TBK1-IRF3 signaling, we infected inhibitor-treated knockout cells with VSV-GFP or vaccinia virus. Depletion of any member of the cGAS-STING-TBK1-IRF3 signaling cascade, which was required for a KDM5 inhibition-triggered interferon response, diminished the antiviral effects of inhibitor treatment, further confirming the requirement of the cGAS-STING-TBK1-IRF3 pathway for KDM5 inhibition-mediated interferon response (Fig 3H and S4A–S4C Fig).

### Inhibition of KDM5 demethylases induces STING expression

**Fig 4:**
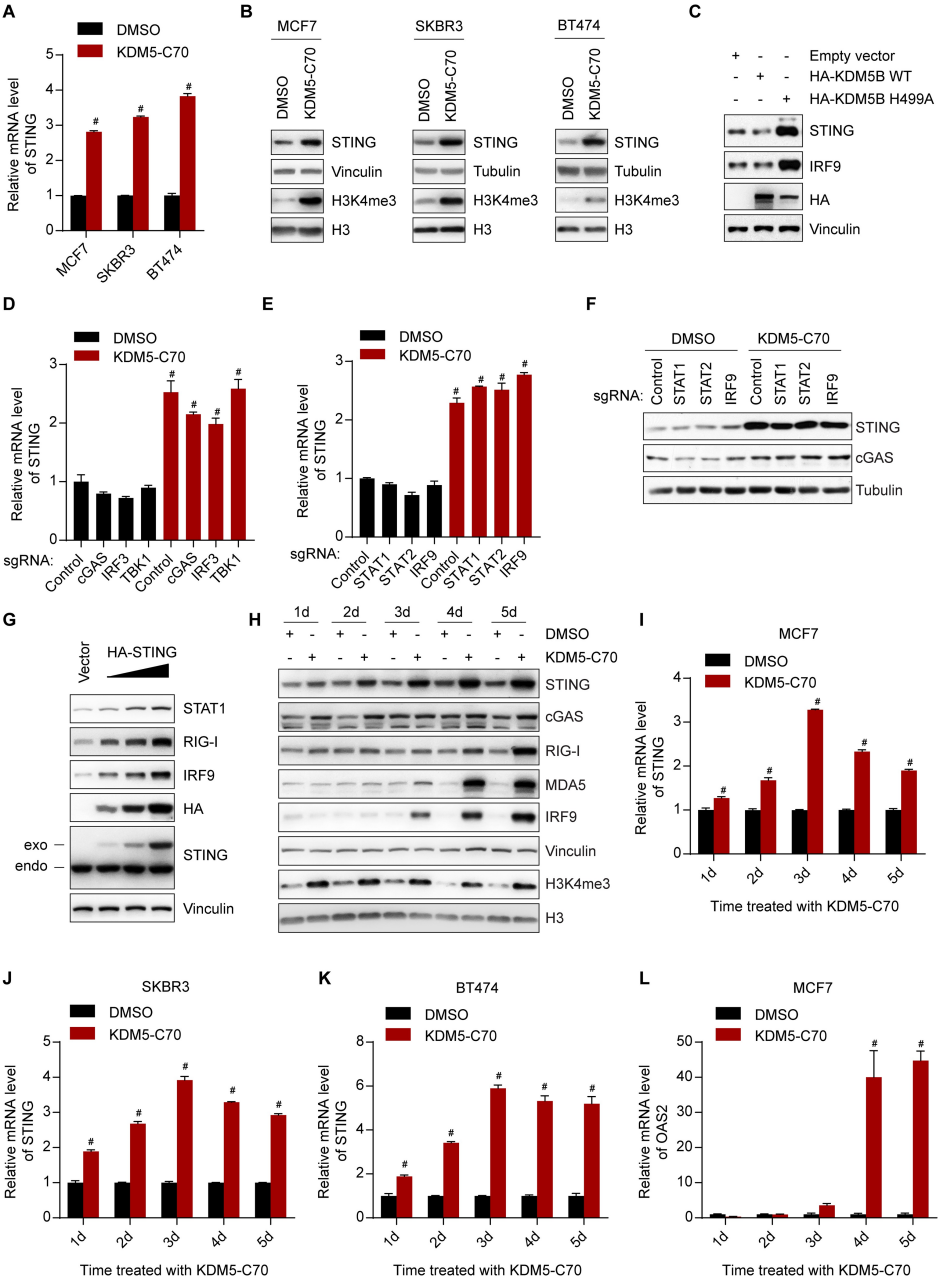
Inhibition of KDM5 demethylases induces STING expression. (A, B) RT-qPCR analysis (panel A) or western blot (panel B) analysis of the indicated cells after treatment with DMSO or 1 μM KDM5-C70 for 3 days. (C) Western blot analysis of MCF7 cells transfected with the indicated expressing plasmids. (D–F) RT-qPCR (panel D and E) or western blot (panel F) analyses of MCF7 cells with knockout of indicated genes after treatment with DMSO or 1 μM KDM5-C70 for 6 days. (G) Western blot analysis of MCF7 cells 2 days after transfection with different amounts of the indicated plasmid. (H–L) Western blot (panel H) or RT-qPCR (panel I–L) analysis of MCF7, SKBR3, or BT474 cells after treatment with DMSO or 1 μM KDM5-C70 for the indicated length of time. Representative data from triplicate experiments are shown. Error bar denotes SEM. ^#^*p* < 0.01 for inhibitors versus DMSO (panel A, D, E, and I–L). The numerical values used to generate graphs in panel A, D, E, and I–L are available in S1 Data. RT-qPCR, reverse transcription followed by quantitative PCR; STING, stimulator of interferon genes.

We showed that the cGAS-STING-TBK1-IRF3 axis was required for KDM5 inhibition-triggered interferon response (Fig 3). The increase of STING after inhibitor treatment does not require IRF3 and TBK1 (Fig 3B), suggesting that STING is directly regulated by KDM5 enzymes in this axis. Both mRNA and protein levels of STING significantly increased after treatment with KDM5-C70 in MCF7, SKBR3, and BT474 breast cancer cells (Fig 4A and 4B) and was variably up-regulated in most of the other cell lines examined (S5A–S5D Fig). Consistently, knockout or knockdown of *KDM5B* and *KDM5C* (S5E–S5H Fig), or overexpression of KDM5B H499A mutant, but not wild-type KDM5B, led to STING increase (Fig 4C). The induction of STING by KDM5 inhibitor treatment or by siRNA-mediated combinatorial knockdown of *KDM5B* and *KDM5C* was not affected by *cGAS*, *IRF3*, *TBK1*, *STAT1*, *STAT2*, or *IRF9* knockout (Figs 3B and 4D–4F and S5H Fig), excluding the possibility that the increase of STING was secondary to an activated interferon response. This is in contrast to the RNA sensors RIG-I and MDA5, whose inhibitor-dependent inductions were attenuated upon *STING*, *cGAS*, *IRF3*, or *TBK1* knockout (Fig 3B). Overexpression of STING in MCF7 cells was sufficient to induce an interferon response (Fig 4G), further supporting that increased STING per se was responsible for the interferon response resulting from KDM5 inhibition.

To further dissect the mechanisms of STING activation and interferon response, we conducted time course studies to examine the effects of KDM5-C70 on H3K4me3 levels and expression levels of STING and ISGs. The global levels of H3K4me3 increased at day 1 after KDM5-C70 treatment and remained high over time (Fig 4H). *STING* mRNA levels began elevating at day 1 and peaked at day 3 in all 3 cell lines (Fig 4I–4K). Consistently, STING protein levels also started to increase at day 1 and further increased over time (Fig 4H). In contrast, the activation of ISGs, including RIG-I, MDA5, IRF9, and *OAS2*, was first seen at day 3 or day 4 (Fig 4H and 4L). Thus, STING induction preceded activation of ISGs, further supporting that STING mediates KDM5 inhibition-induced interferon response.

### KDM5B and KDM5C bind to the promoter of *STING* and directly suppress *STING* expression

**Fig 5:**
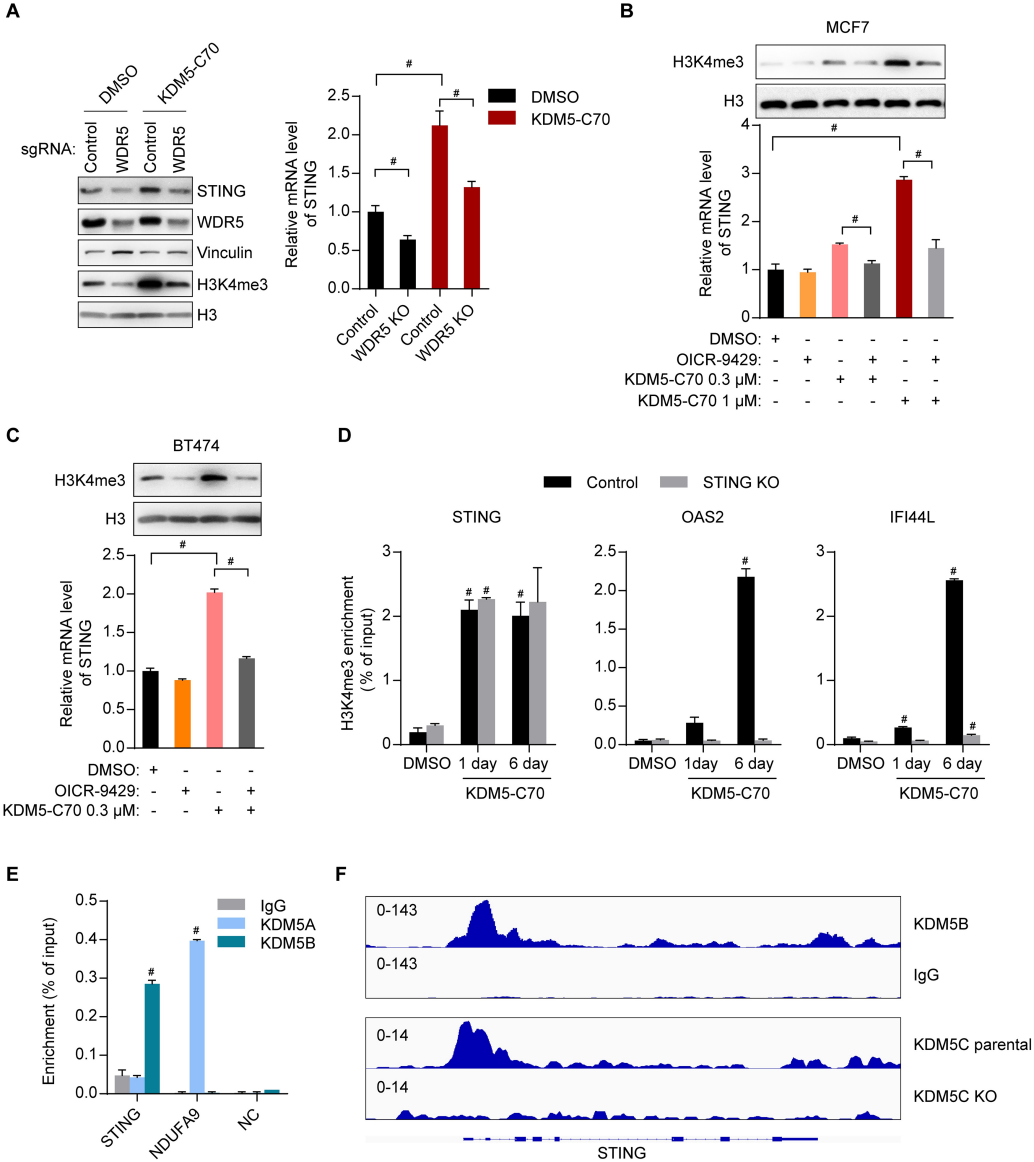
KDM5B and KDM5C bind to the promoter of *STING* and directly suppress *STING* expression. (A) Western blot (left panel) and RT-qPCR (right panel) analyses of STING in MCF7 cells with knockout of the indicated genes after treatment with DMSO or 1 μM KDM5-C70 for 3 days. (B, C) Western blot analysis of H3K4me3 (upper panel) and RT-qPCR analysis of *STING* (lower panel) in MCF7 cells (panel B) or in BT474 cells (panel C) after treatment with the indicated compounds for 3 days. The concentration of OICR-9429 was 20 μM. (D) H3K4me3 ChIP-qPCR analysis at the promoter of *STING*, *OAS2*, or *IFI44L* in control or STING knockout MCF7 cells treated with DMSO or 1 μM KDM5-C70 for 1 day or 6 days. (E) KDM5A and KDM5B ChIP-qPCR analysis of MCF7 cells at the *STING* and *NDUFA9* promoters, or downstream of the last *STING* exon as NC. (F) Analysis of ChIP-seq data for KDM5B binding at the *STING* genomic region in K562 cells (GSE29611, upper panel) and KDM5C in ZR-75-30 cells (GSE71327, lower panel) [42]. Representative data from triplicate experiments are shown. Error bar denotes SEM. ^#^*p* < 0.01 for the comparisons shown in panel A–C, for inhibitors versus DMSO (panel D), and for KDM5A or KDM5B ChIP versus IgG ChIP (panel E). The numerical values used to generate graphs in panel A–E are available in S1 Data. ChIP, chromatin immunoprecipitation; ChIP-seq, chromatin immunoprecipitation sequencing; IgG, immunoglobulin G; NC, negative control; RT-qPCR, RT-qPCR, reverse transcription followed by quantitative PCR; STING, stimulator of interferon genes.

We next asked whether decreasing the level of H3K4me3, the KDM5 substrate, affects STING expression. The WD40-repeat protein WDR5 is a core component of H3K4 methyltransferase complexes and critical for tri-methylation of H3K4 [40]. Both *WDR5* knockout or WDR5 inhibitor OICR-9429, which prevents the binding of WDR5 to the methyltransferase complexes [41], precluded H3K4me3 increase by KDM5 inhibition and abolished the effect of KDM5 inhibition on STING expression (Fig 5A–5C). In addition, chromatin immunoprecipitation (ChIP)-qPCR analysis showed that H3K4me3 at the promoter of *STING* is induced by KDM5 inhibitor treatment for 1 day in both MCF7 (S6A Fig) and BT474 cells (S6B Fig). In contrast, treatment by KDM5-C70 inhibitor for 1 day had minimal effects on H3K4me3 at the promoters of *GAPDH* and *IFNβ* (S6A and S6B Fig). Although H3K4me3 at the promoter of ISGs such as *OAS2* and *IFI44L* increased at day 1, their increases were much smaller than those at day 6 (Fig 5D). These increases of H3K4me3 were abolished in *STING* knockout cells (Fig 5D), consistent with the idea that KDM5 loss-triggered interferon response results from increased H4K3me3 at the *STING* promoter and the subsequent up-regulation of STING. Furthermore, KDM5B binds to the promoter of *STING* in MCF7 cells (Fig 5E) and K562 cells (Fig 5F), while KDM5C binds to the promoter of *STING* in ZR-75-30 cells (Fig 5F). In contrast, KDM5B and KDM5C do not directly bind to the promoter of *cGAS* or downstream ISGs, such as *OAS2*, *IFI44L*, and *IFI44* (S6C Fig). In comparison, although KDM5A binds to the promoter of a known KDM5A target *NDUFA9* [29], it does not bind to the *STING* promoter (Fig 5E). These data suggest that KDM5B and KDM5C maintain a low level of H3K4me3 at the *STING* promoter, suppress *STING* expression, and prevent the STING-mediated interferon response.

### KDM5 inhibition induces a robust interferon response only in cancer cells with elevated levels of cytosolic DNA

**Fig 6:**
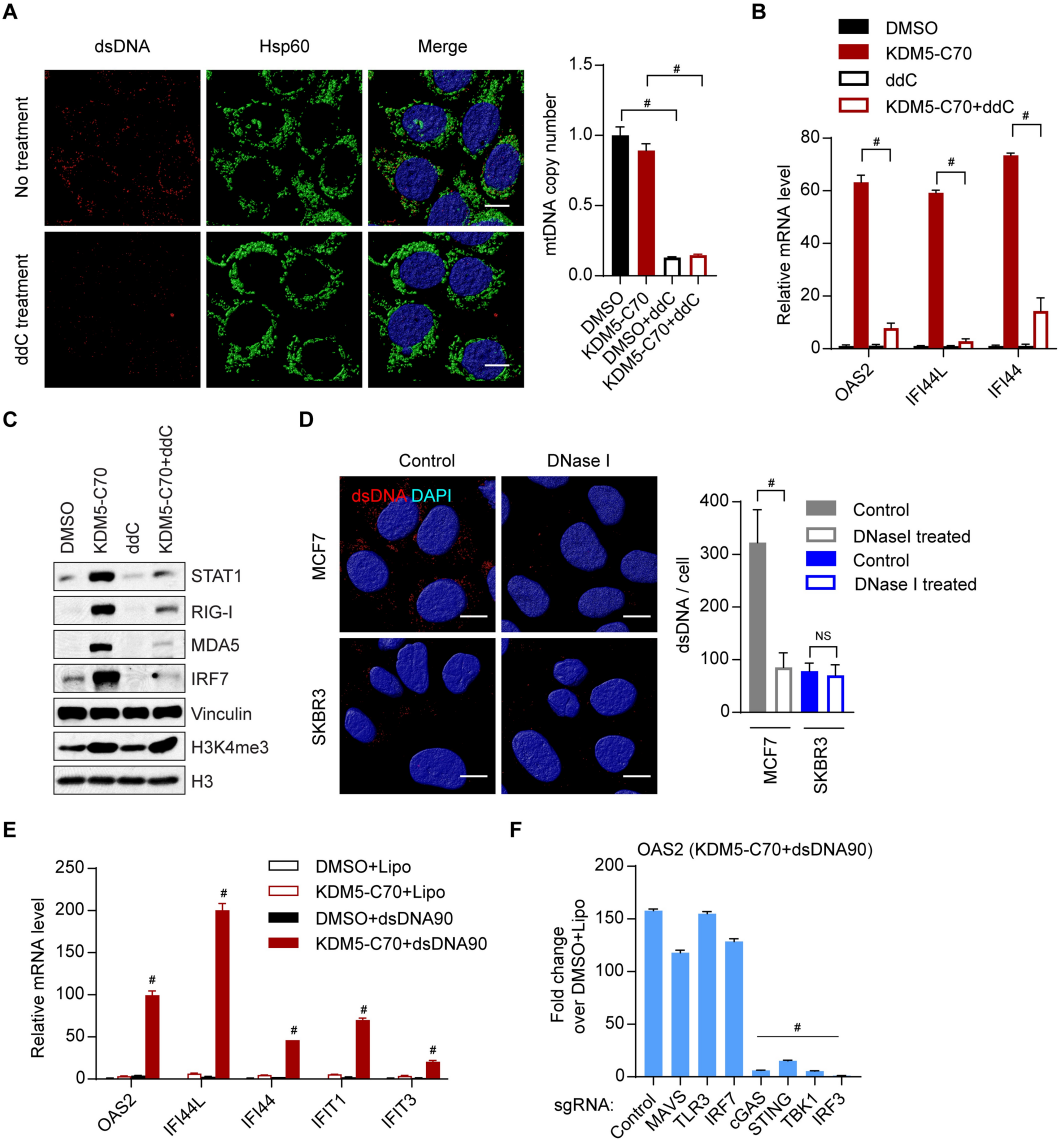
KDM5 inhibition induces a robust interferon response in cancer cells with elevated levels of cytosolic DNA. (A) Immunostaining of dsDNA and the mitochondrial marker Hsp60 in MCF7 cells. Surface plots of Z-stack images generated with Huygens (left panel). qPCR detecting mtDNA copy number in cells with the indicated treatment (right panel). 10 μM of ddC was used. Scale bar, 10 μm. (B, C) RT-qPCR (panel B) and western blot (panel C) analyses of MCF7 cells with the indicated treatment. MCF7 cells were treated with 10 μM ddC and 1 μM KDM5-C70 for 6 days. Cells were refed every 2 days. (D) dsDNA and DAPI staining of MCF7 cells or SKBR3 cells digested with 50 μg/ml DNase I. Surface plots of Z-stack images generated with Huygens (left panel). Quantification of dsDNA intensity per cell using image J was shown in the right panel. Scale bar, 10 μm. (E) RT-qPCR analyses of SKBR3 cells with the indicated treatment. SKBR3 cells were transfected with 1 μg dsDNA of about 90 bp (dsDNA90) using Lipofectamine 2000 5 hours before treatment with DMSO or 1 μM KDM5-C70 for 3 days. (F) RT-qPCR analysis of SKBR3 cells with knockout of the indicated genes after treatment with 1 μg dsDNA and 1 μM KDM5-C70 for 3 days. Representative data from triplicate experiments are shown. Error bar denotes SEM. ^#^*p* < 0.01 for panel A, B, and D–F, for KDM5-C70 + dsDNA90 versus DMSO + lipo (panel E), and for knockout sgRNA versus control sgRNA (panel F). The numerical values used to generate graphs in panel A, B, and D–F are available in S1 Data. ddc, dideoxycytidine; dsDNA, double-stranded DNA; lipo, Lipofectamine 2000; mtDNA, mitochondrial DNA; RT-qPCR, RT-qPCR, reverse transcription followed by quantitative PCR; sgRNA, single guide RNA.

We noticed that overexpression of STING was sufficient to trigger a robust interferon response in MCF7 cells (Fig 4G), but knockout of cGAS blocked the induction of interferon response by KDM5 inhibition in these cells (Fig 3B and 3D). These data suggested that MCF7 cells had sufficient cytosolic DNA to bind cGAS and trigger cGAMP production to activate STING but had a low level of STING protein that prevented a robust interferon response. Tumor cytosolic DNA can be derived from mitochondria, nuclear DNA leakage, micro-nuclei, or other sources such as oncoviruses [43–48]. We first examined whether MCF7 cells have cytosolic DNA. MCF7 cells were costained with dsDNA and the mitochondrial marker Hsp60. As expected, we observed dsDNA in the cytoplasm of MCF7 cells, but most of these dsDNA did not colocalize with mitochondria (Fig 6A). Treatment with dideoxycytidine (ddC), a deoxyribonucleoside analogue that specifically inhibits mitochondrial DNA (mtDNA) replication [6, 46], led to a dramatic decrease of mtDNA (Fig 6A, right panel) and disappearance of cytosolic DNA (Fig 6A, left panel). These results indicated that cytosolic DNA in MCF7 is mainly derived from mitochondria. To test the requirement of cytosolic DNA derived from mitochondria for the induction of interferon response by KDM5 inhibition, we treated MCF7 cells with KDM5-C70 and ddC. Treatment of ddC strongly inhibited the induction of ISGs by KDM5-C70 (Fig 6B and 6C). These results suggest that mtDNA is required for KDM5-inhibition–triggered interferon response in MCF7 cells. In contrast, treatment with leptomycin B (LMB), an inhibitor of nuclear DNA export, prevented the induction of ISGs by Ataxia-telangiectasia mutated (ATM) and Ataxia-telangiectasia and Rad3-related protein (ATR) inhibitor VE-821 treatment (S7A Fig) [49, 50] but did not suppress the ISG induction by KDM5 inhibition (S7B Fig). These results indicate that nuclear DNA leakage is not the major source of cytosolic DNA in MCF7 cells. Further experiments will be necessary to exclude the possibility that nonmitochondria-derived sources of cytosolic DNA contribute to ISG induction. It is worth mentioning that KDM5 inhibitor treatment did not alter the amount of cytosolic DNA in these cells (S7C Fig). In contrast to MCF7 cells, we observed limited cytosolic DNA in SKBR3 cells (Fig 6D), in which the induction of interferon response by KDM5 inhibition was less robust compared with MCF7 cells (S2D Fig), suggesting that the amount of cytosolic DNA is also a limiting factor for a potent interferon response. To further examine this possibility, we introduced additional cytosolic DNA into SKBR3 cells by transfecting dsDNA, and followed with KDM5-C70 treatment. Treatment with dsDNA or KDM5-C70 alone only led to minimal increase of ISGs, while combinatorial treatment with dsDNA and KDM5-C70 dramatically induced ISGs (Fig 6E). This induction was blocked by knockout of cGAS, STING, TBK1, or IRF3 (Fig 6F and S7D Fig). These data demonstrate that cytosolic DNA is required for full activation of interferon response upon KDM5 inhibition, suggesting that cancer cells with an elevated level of cytosolic DNA can elicit a strong interferon response upon STING induction by KDM5 loss or inhibition.

### KDM5B expression is negatively correlated with STING expression, T-cell infiltration, and patient survival

**Fig 7:**
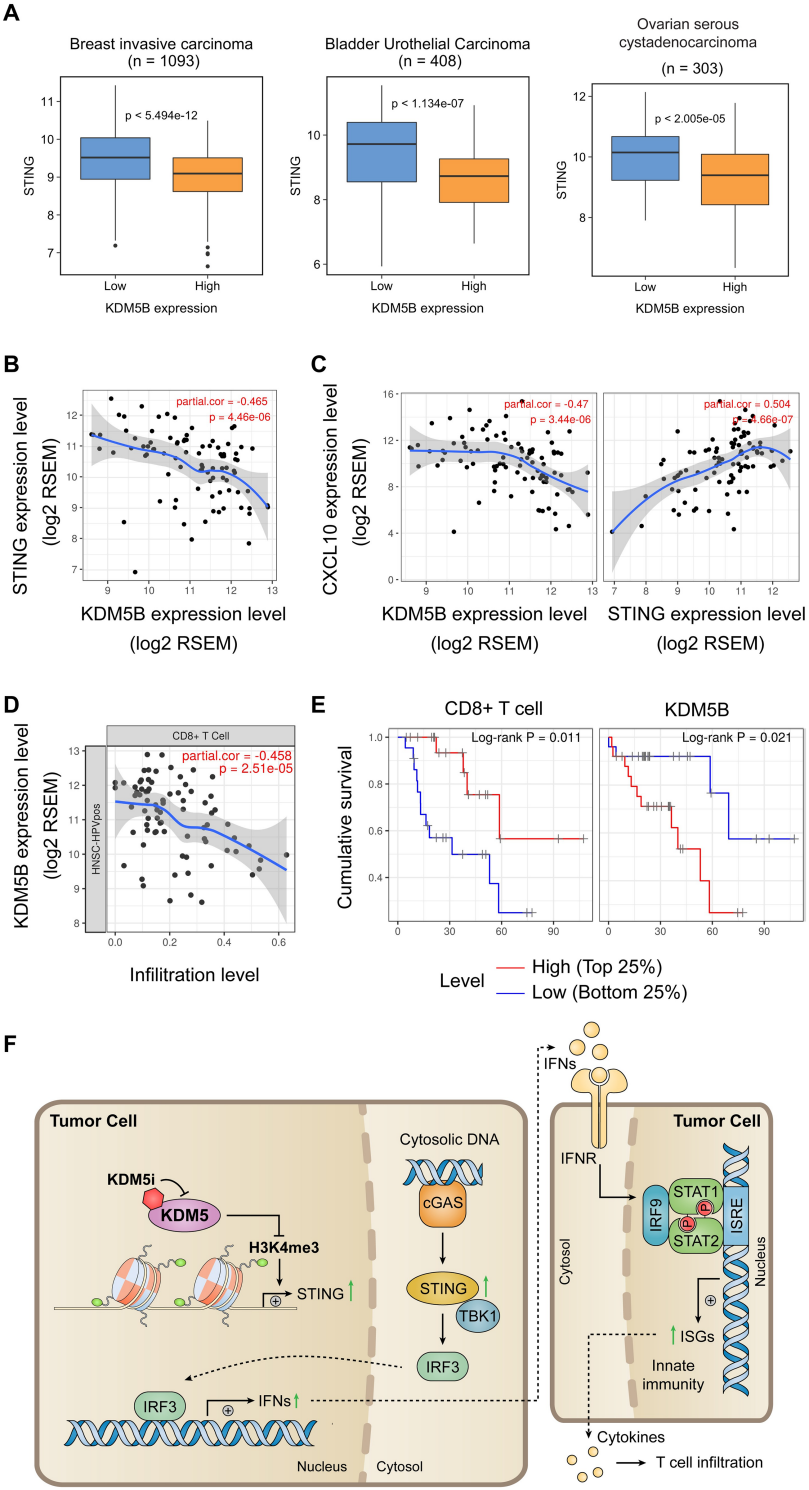
KDM5B is negatively associated with STING expression, CD8^+^ T-cell infiltration, and clinical outcome. (A) Anticorrelation between expression of KDM5B and STING in TCGA cancer samples. Normalized STING levels in “KDM5B low” and “KDM5B high” samples of breast invasive carcinoma, bladder urothelial carcinoma, and ovarian serous cystadenocarcinoma from the TCGA datasets. The numerical values used to generate these graphs are available in S1 Data. (B) Correlation between KDM5B and STING in HPV^+^ head and neck tumors. *n* = 79. (C) Correlation between KDM5B and CXCL10 (left panel) or STING and CXCL10 (right panel) in HPV^+^ head and neck tumors. (D) Correlation between KDM5B expression and CD8^+^ T-cell infiltration in HNSC-HPVpos. (E) Association of CD8^+^ T-cell infiltration level (left panel) and KDM5B expression (right panel) with survival of HPV^+^ head and neck cancer patients. Tumors in the top 25th percentile were compared to those in the bottom 25th percentile. (F) A working model for how KDM5i induces innate and adaptive immune responses. HPV, human papilloma virus; HNSC-HPVpos, HPV^+^ head and neck tumors; ISRE, interferon-stimulated response element; KDM5i, KDM5 inhibitor; STING, stimulator of interferon genes; TCGA, The Cancer Genome Atlas.

To validate the regulation of STING by KDM5 in human patients, we compared STING expression levels in “KDM5B low” and “KDM5B high” samples. We found that STING expression level is lower in “KDM5B high” samples than in “KDM5B low” samples from multiple human tumor types, including breast invasive carcinoma, bladder urothelial carcinoma, and ovarian serous cystadenocarcinoma (Fig 7A). To validate the effects of KDM5 on interferon response in tumors with an elevated level of cytosolic DNA, we analyzed human papilloma virus (HPV; a dsDNA oncovirus)-induced tumors, such as head and neck cancer and cervical cancer. In HPV^+^ head and neck cancer, we found significant negative correlation between KDM5B and STING expression, with a Spearman’s correlation of −0.465 (Fig 7B). Despite the inability to separate HPV^+^ and HPV^−^ cervical cancer, we observed significant negative correlation between KDM5B and STING expression in cervical cancer, with a Spearman’s correlation of −0.172 (S8A Fig). CXCL10 is one of the interferon-stimulated chemokines that promotes infiltration of immune cells into the tumor microenvironment [10, 51]. We found CXCL10 expression negatively correlated with KDM5B expression in HPV^+^ head and neck cancer and positively correlated with STING expression in both HPV^+^ head and neck cancer and cervical cancer (Fig 7C and S8B Fig). Additionally, we found that CD8^+^ T-cell infiltration was negatively associated with KDM5B, especially in HPV^+^ head and neck cancer (correlation score −0.458) (Fig 7D and S8C Fig). Lastly, we found a positive correlation between CD8^+^ T-cell infiltration level and patient survival and a negative correlation between KDM5B expression and patient survival in HPV^+^ head and neck cancer (Fig 7E). These data show that tumors with high KDM5B expression levels present with low STING expression, suppressed interferon response, and decreased tumor-infiltrating lymphocytes, especially in the presence of abundant cytosolic DNA. As a result, high KDM5B expression is associated with poor prognosis, suggesting KDM5B as a potential target of immunotherapy.

## Discussion

Here, we identified a novel epigenetic regulatory mechanism that tumor cells use to avoid damage caused by cytosolic DNA-triggered innate immune response. Specifically, expression of *STING*, a key component of the interferon pathway, was silenced by KDM5 family demethylases through removal of H3K4me3 from the *STING* locus. Suppression of *STING* by KDM5 demethylase blocked the signal transduction initiated by cytosolic DNA and mediated by the cGAS-STING-TBK1-IRF3 axis (Fig 7F). Inhibition or depletion of KDM5B and KDM5C—by small-molecule inhibitors, siRNA-mediated knockdown, or CRISPR/Cas9-mediated knockout—enhanced STING expression and activated ISGs. The enhanced STING expression was dependent on the activity of H3K4 methyltransferases. This epigenetic regulation allows for a fast, robust, and reversible control of the interferon pathway and is thus expected to have major implications in controlling infection by DNA-containing pathogens and treating cancer.

Robust activation of the cGAS/STING pathway requires not only STING activation by cGAMP—generated by cGAS after it binds pathogen-derived or abnormal self-DNA in the cytosol—but also sufficient STING protein to mediate the signal cascade. Although cytosolic DNA is commonly found in tumor cells [44, 52–56], cGAS-STING signaling is disrupted or silenced in many tumors, enabling cancer cells to evade immunosurveillance [12–15]. Recent studies showed that the expression levels of *cGAS* and *STING* were inversely correlated with DNA methylation and can be activated by a DNA methyltransferase (DNMT) inhibitor in a subset of colorectal cancer and melanoma cells [12–14], indicating that DNA methylation contributes to silencing of the cGAS-STING pathway. Here, we found that *STING* was up-regulated by KDM5 inhibitors in a panel of cell lines, and the expression levels of KDM5B and STING were negatively associated in multiple tumor datasets. These results suggest that regulation of *STING* by KDM5 is another common mechanism to modulate the cGAS/STING pathway.

Epigenetic changes contribute to tumorigenesis through reprogramming of gene expression profiles [57]. Alternations of epigenetic marks, caused by dysregulation of their writers and erasers, are reversible [58]. This makes epigenetic regulators very attractive drug targets. In fact, inhibitors of epigenetic regulators are either approved or under extensive clinical development, such as inhibitors against DNMTs, Enhancer of zeste homolog 2 (EZH2), histone deacetylases (HDACs), and bromodomain proteins. Emerging evidence shows that, in addition to their effects on tumor cells, these inhibitors also affect the tumor microenvironment, including immune cells [59]. Previous studies, including ours, have shown that KDM5 family histone demethylases, especially KDM5A and KDM5B, are highly expressed and promote tumorigenesis in multiple cancer types [17, 21–29]. The mechanisms for their up-regulation in cancer remain largely unknown. KDM5B was identified as a gene up-regulated by HER2 in human breast cancers [19]. KDM5B undergoes post-translational modifications such as SUMOylation by small ubiquitin-like modifier protein (SUMO) E3 ligase hPc2 and ubiquitination by ubiquitin E3 ligase RNF4 that mediates KDM5B for proteasomal degradation [60]. KDM5B and KDM5C are also regulated by microRNA (miRNA)-137 and miRNA-138, respectively. Both miRNAs are down-regulated in several breast cancer cell lines compared with nontumorigenic human mammary epithelial cell line MCF10A, consistent with the higher expression levels of KDM5B and KDM5C in these cancer cells [61]. In line with the oncogenic roles of KDM5A and KDM5B, suppression of KDM5A or KDM5B delays tumor formation, metastasis, and drug resistance in breast, lung, melanoma, and gastric cancers [17, 21–29]. Although inhibition of KDM5C could have adverse effects on neuronal circuits [62] or promote tumor formation in clear cell renal carcinoma [63] and cervical cancer [64], KDM5C was also shown to have oncogenic roles in prostate cancer [65].

Small-molecule inhibitors of KDM5 enzymes have been developed for cancer treatment [30, 33, 34, 66, 67]. Here, we find that KDM5 inhibitors trigger a robust interferon response through a STING-dependent manner. Further development of these inhibitors could lead to a new class of cancer immunotherapeutic drugs. The cGAS/STING pathway has been targeted in the clinic to induce both innate immune response and subsequent adaptive immune response for cancer treatment. Small-molecule agonists of STING induce systemic immune responses and regression of established tumors in mice [10, 68]. However, this strategy is predicted to have limited efficacy in tumors with abnormal cytosolic DNA but silenced STING. In these tumors, such as HPV^+^ head and neck or cervical tumors, KDM5 inhibitors could be used to restore STING expression and induce antitumor immune responses. Furthermore, while immune checkpoint inhibitors have achieved remarkable success, most patients do not respond to these treatments. A major mechanism of intrinsic resistance to these treatments is due to lack of T-cell infiltration, which could be induced by STING activation. In fact, inhibition of the cGAS/STING pathway prevents the therapeutic effects of immune checkpoint blockade in a mouse model [69]. Therefore, KDM5 inhibitors, or a combination of STING agonists and KDM5 inhibitors, could maximize the antitumor immune response and allow for effective treatment of nonresponders to the current immunotherapies.

## Materials and methods

### Antibodies and reagents

Antibody for KDM5A was described previously [26]. The following antibodies were obtained commercially: rabbit anti-histone H3 (ab1791) (Abcam, Cambridge, UK); rabbit anti-KDM5B (HPA027179) (Sigma, St. Louis, MO); mouse anti-tubulin (T5168) (Sigma, St. Louis, MO); mouse anti-STAT1 (sc-345), −STAT2 (sc-514193), and −IRF9 (sc-135953) (Santa Cruz, Dallas, TX); goat anti-Hsp60 (sc-1052) (Santa Cruz, Dallas, TX); rabbit anti-KDM5C (A301-034A) (Bethyl, Montgomery, TX); rabbit anti-H3K4me3 (C42D8), −H3K4me1 (D1A9), −H3K4me2 (C64G9), −H3K9me3 (D4W1U), −H3K27me3 (C36B11), −H3K36me3 (D5A7), −RIG-I (D14G6), −MDA5 (D74E4), −STING (D2P2F), −cGAS (D1D3G), −IRF3 (D83B9), −TBK1 (D1B4), −MAVS (3993), −IRF7 (4920), −Phospho-STAT1 (58D6), and −HA (C29F4) (Cell Signaling Technology, Danvers, MA); and mouse anti-dsDNA (MAB1293) (Millipore, Burlington, MA).

pcDNA3.1-3xHA-KDM5B construct was described previously [66]. An H499A mutation was introduced into KDM5B plasmid by site-directed mutagenesis. pcDNA3.1-3xHA-STING construct was generated by PCR amplification of the full length of STING coding sequence from cDNA and inserting into pcDNA3.1-3xHA vector between BamHI and XhoI sites. Compound OICR-9429, VE821 was purchased from Sigma. LMB was purchased from Santa Cruz (Dallas, TX) (sc-202210). KDM5-C70 (NCGC00371443) was purchased from Xcess Biosciences (San Diego, CA). Compounds Dong-A-167 (NCGC00487054), GDC-50 (NCGC00482457) [33], and CPI-48 (NCGC00488278) [34] were prepared according to patents WO2016/68580, WO2016/57924, and WO2015/135094, respectively.

### Crystallography of inhibitor CPI-48 in complex with KDM5A catalytic domain

The linked KDM5A JmjN-JmjC catalytic domain was prepared and purified by 3-column chromatography utilizing affinity, anion exchange, and sizing exclusion as previously described in detail [70]. The purified protein, in 20 mM Hepes (pH 8.0), 300 mM NaCl, 5% glycerol, and 0.5 mM tris (2-carboxyethyl)phosphine (TCEP), was mixed with MnCl_2_ and αKG at an approximate molar ratio of 1:5 and concentrated to approximately 10 mg/ml (280 μM) for co-crystallization as described [30]. Inhibitor CPI-48 was soaked into these preformed crystals of KDM5A-αKG-Mn(II) complexes by transferring a crystal into a new drop containing mother liquor (1.2–1.35 M [NH4]_2_SO_4_, 0.1 M Tris-HCl [pH 8.6–9.2], 0%–20% glycerol, and 25 mM [Na/K] dibasic/monobasic phosphate) and CPI-48 (approximately 500 μM), allowing the crystal to remain in this drop overnight for CPI-48 to exchange with αKG. The crystals were then mounted into nylon cryoloops (Hampton Research, Aliso Viejo, CA) and frozen in liquid nitrogen after the addition of more glycerol (up to approximately 30% total) to the mother liquor as a cryoprotectant. X-ray diffraction data were collected SER-CAT beam-line 22-ID at the Advanced Photon Source at Argonne National Laboratory at 100 K with 1-degree oscillation images, and the structure was determined by molecular replacement and refinement performed as described (S1 Table) [30].

### AlphaLISA-based demethylase assays

AlphaLISA assays were performed and analyzed as described previously [30] with 25 nM KDM5A (BPS Biosciences, San Diego, CA; 50110), 10 nM KDM5B (1–755) ΔAP [70], 20 nM KDM5B [30, 66], or 25 nM KDM5C (BPS Biosciences, San Diego, CA; 50112).

### Cell culture, transfection, and histone extraction

MCF7 and BT474 cells were cultured in RPMI1640 supplemented with 10% fetal bovine serum and 1% penicillin and streptomycin. SKBR3 cells were cultured in Dulbecco’s Modified Eagle Medium supplemented with 10% fetal bovine serum and 1% penicillin and streptomycin. siRNA transfections were performed using RNAiMAX (Invitrogen), and plasmid transfections were performed using Lipofectamine 3000 (Invitrogen) according to the manufacturer’s instructions. The sequence of dsDNA90 was described previously [71], transfected using Lipofectamine 2000 (Invitrogen) according to the manufacturer’s instructions. siRNA universal negative control 1 and 2 were purchased from Sigma (SIC001 and SIC002). siKDM5A targeting sequences were described previously [29]. Other siRNA targeting sequences were as follows: siKDM5B-1, CAGTGAATGAGCTCCGGCA; siKDM5B-2, GGAGCTGACATTGCCTCAA; siKDM5C-1, GGAGGAAGGTGGTTATGAA; and siKDM5C-2, GGAGGAAGGTGGTTATGAA.

Histone extraction was conducted as described previously [66].

### CRISPR/Cas9-mediated knockout

sgRNAs were designed using CHOPCHOP (https://chopchop.rc.fas.harvard.edu/) and cloned into LentiCRISPRv2. Knockout cells were generated as described previously [31]. Briefly, 293T cells in 6-well plates were introduced with 1.5 μg lentiviral plasmid, 1 μg psPAX2, and 0.5 μg pMD2.G. At 48 hours after transfection, lentivirus-containing media were collected and filtered through a 0.45 μm filter before being used to infect cells. Cells were infected with lentivirus for 24 hours, then refed with fresh medium with puromycin. sgRNA controls were described previously [67]. Other sgRNA targeting sequences are listed in S2 Table.

T7 endonuclease assays were conducted as described previously [31]. The primers for amplifying the region flanking TLR3 sgRNA targeting site were as follows: TLR3-F, TCATGAGACAGACTTTGCCTTG; and TLR3-R, GGCTATACCTTGTGAAGTTGGC.

### Viral stocks and infections

Vaccinia viruses are recombinant vaccinia virus (vTF7-3, strain WR) expressing T7 RNA polymerase [72]. They were kindly provided by Linda Buonacore and Dr. John Rose (Yale University, New Haven, CT). VSV-GFP viruses (VSV-G/GFP, Indiana strain) were generated as described previously [73]. MCF7 cells were infected and incubated at MOI indicated in the figure legends for the indicated time. FACS analyses were performed using a Stratedigm 13-color cytometer with cells fixed in 4% paraformaldehyde. FACS plots were first gated on live cells before analyzing viral GFP fluorescence. Viral copy numbers of vaccinia virus were determined by quantification of pox14KD [74].

### Immunostaining and imaging

For immunostaining, cells were seeded on coverslips, fixed with 4% paraformaldehyde for 10 minutes, permeabilized with 0.4% Triton in PBS for 5 minutes, and then blocked with 10% FBS before incubation with primary antibodies at 4°C overnight. dsDNA staining and image processing were performed according to previous studies [54, 55]. For DNase I–treated samples, cells were permeabilized with 10 μg/ml digitonin and 50 μg/ml DNase I for 30 minutes at 37 °C before fixation with 4% paraformaldehyde. Z-stack images were taken using Leica SP5 confocal microscope. Surface rendering of 3D Z-stacks were processed using Huygens with threshold levels set based on DNase I–treated samples.

### ChIP-qPCR and RT-qPCR

ChIP assays were conducted as described previously [75]. Total RNA was isolated using RNeasy Plus Mini Kit (Qiagen, Hilden, Germany). Reverse transcription was performed using High-Capacity cDNA Reverse Transcription Kit (ABI, Sterling, VA). For both ChIP-qPCR and RT-qPCR, qPCR analyses were performed in triplicate using Fast SYBR Green Master Mix (Applied Biosystems, Foster City, CA).

The primers for RT-qPCR analysis of *ISG15*, *RIG-I*, *MDA5*, *IFNβ*, *IFNλ1*, and *IFNλ2* were described previously [76]. Other primers for RT-qPCR are listed in S3 Table. The primers for ChIP-qPCR are listed in S4 Table.

### RNA-seq and bioinformatic analysis

MCF7 cells were treated with 3 μM of KDM5-C70 or CPI-48 for 6 days. Total RNA was isolated using RNeasy Plus Mini Kit (Qiagen, Hilden, Germany). mRNA libraries for sequencing were prepared according to the standard Illumina protocol. Sequencing (100 bp, paired-end) was performed using Illumina HiSeq 2000 sequencing system at the Genomics Core of Yale Stem Cell Center. RNA-seq data were deposited in the National Center for Biotechnology Information (NCBI) Gene Expression Omnibus database under accession number GSE108502.

The RNA-seq reads were mapped to human genome (hg38) with Bowtie2 [77] in local mode, which allows the reads spanning the exon–exon junctions to get mapped to one of the 2 exons (whichever gives the higher mapping score) independent of the transcriptome annotation. The uniquely mapped reads (cutoff: MAPQ >10) were counted to ENCODE gene annotation (version 24) [78] using FeatureCounts [79]. Differential gene expression was performed with DESeq2 [80].

Gene expression profiles of DMSO- or KDM5-inhibitor–treated cells were used for GSEA using GSEA version 2.0 software [81]. The gene set database of h.all.v6.1.symbols.gmt (Hallmarks) was used. Statistical significance was assessed by comparing the enrichment score to enrichment results generated from 10,000 random permutations of the gene set.

### Analysis of TCGA datasets

TCGA expression datasets were downloaded using the Broad Institute Firehose application programming interface (https://gdac.broadinstitute.org). Expression data are in log2 RSEM format. For each TCGA dataset, primary tumor samples were ranked by their expression of KDM5B and evenly divided into 4 groups. Samples with KDM5B expression less than the first quartile were deemed “KDM5B low,” while samples with KDM5B expression greater than or equal to the third quartile were deemed “KDM5B high.” Statistical comparisons were performed between the STING expression of the samples in “KDM5B low” and “KDM5B high” groups. Significance was computed using the Student *t* test. For box plots, the lower and upper hinges signify the first and third quartiles, respectively, while the center line depicts the median. The whisker tips correspond to the first observation beyond 1.5 times the interquartile range. Outliers are illustrated with points. R scripts are available upon request. The correlation between KDM5B and clinical impact in HPV-positive head and neck cancer or cervical cancer were analyzed using a web server TIMER (https://cistrome.shinyapps.io/timer/) [82, 83]. The correlation between KDM5B and STING, KDM5B and CXCL10, or STING and CXCL10 were adjusted by tumor purity.

### Visualization of KDM5B and KDM5C ChIP-seq

KDM5B and input ChIP-seq data were obtained from the ENCODE K562 dataset (GSE29611) in bigwig format. KDM5C wild-type and knockout ChIP-seq data were obtained from GSE71327 [42], aligned with Bowtie2, and processed into bigwig using Deeptools [84]. All signal tracks were visualized using IGV [85].

### Statistical analysis

Statistical significance was determined using the unpaired Student *t* test. Error bars represent SEM. SEM was calculated from triplicate technical replicates of each biological sample or 2 or 3 biological replicates. Data shown were representative of 3 independent experiments or biological replicates as indicated in figure legends.

## Supporting information

S1 Fig KDM5-C70, Dong-A 167, GDC-50, and CPI-48 are potent KDM5 demethylase inhibitors that bind in the active site of KDM5A. (A) KDM5-C70 was designed as a cell-permeable prodrug that is hydrolyzed by intracellular esterase(s) to generate KDM5-C49, which contains an isonicotinic acid moiety with a carboxylic acid (PCT WO 2014053491) [30, 32]. (B) KDM5-C49 binds in the active site pocket bridging between the metal binding site (magenta) and the hydrogen-bonding network mediated by K501-N575-Y409 (PDB 5ISL) [30]. (C, D) A computer model of Dong-A-167 compound bound in the active site of KDM5A (panel C), based on KDM5B in complex with a related compound containing a pyrido[3,4-*d*]pyrimidin-4(1*H*)-one moiety (PDB 5FPL) (D) [86]. (E) GDC-50 (also known as Compound N54) bound in the active site of KDM5A (PDB 6BH2) [36]. (F) Compound CPI-48 bound in the active site of KDM5A. Like GDC-50, Y409 undergoes a conformational change upon the binding of inhibitor, resulting in a van der Waals contact with the isopropyl moiety of CPI-48 (indicated by a red arrow). Crystallographic data of KDM5A-CPI-48 complex in the presence of Mn(II) are shown in S1 Table. (G) AlphaLISA assays showing that KDM5-C70, Dong-A-167, GDC-50, and CPI-48 are potent inhibitors against truncated KDM5B. Shown were representative dose response curves (upper panel) and IC_50_ values (mean +/− SD) (lower panel) of all 4 compounds against KDM5B (1–755) ΔAP from 3 independent experiments. (H) AlphaLISA assays showing that Dong-A-167 is a potent inhibitor of KDM5A, KDM5B, and KDM5C. Shown were representative dose response curves (upper panel) and IC_50_ values (mean +/− SD) (lower panel) of Dong-A-167 against KDM5 demethylases from 3 independent experiments. The numerical values used to generate graphs in panel G and H are available in S1 Data. IC_50_, half maximal inhibitory concentration.
(TIF)

S2 Fig Inhibition of KDM5 activates ISGs. (A) Western blot analyses of MCF7 cells treated with 1 μM KDM5-C70 for 3 days. (B) Pathways that were up-regulated by 3 μM KDM5-C70 or CPI-48 treatment, revealed by GSEA. The gene set database of h. all. v6.1. symbols. gmt (Hallmarks) was used. All the up-regulated pathways in inhibitor-treated cells with FDR q value < 0.05 were shown. (C) RT-qPCR analysis of MCF7 cells treated with 1 μM KDM5-C70, 10 μM each of Dong-A-167, GDC-50, or CPI-48 for 6 days. (D, E) RT-qPCR analysis of SKBR3 cells treated with 5 μM KDM5-C70 (panel D) and BT474 cells treated with 1 μM KDM5-C70 (panel E) for 6 days. (F, G) Western blot (panel F) and RT-qPCR (panel G) analyses of MCF7 cells 5 days after transfection with the indicated siRNAs. KDM5BC, 2 siRNAs targeting KDM5B and KDM5C. Representative data from triplicate experiments are shown. Error bar denotes SEM. ^#^*p* < 0.01 for inhibitors versus DMSO (panel C–E), for KDM5 siRNA versus average of 2 control siRNAs (panel G). The numerical values used to generate graphs in panel C–E and G are available in S1 Data. Control, universal negative control; FDR q value, false discovery rate q value; GSEA, gene set enrichment analysis; NES, normalized enrichment score; NS, nonspecific band; NOM p value, nominal *p*-value; RT-qPCR, RT-qPCR, reverse transcription followed by quantitative PCR; siRNA, small interfering RNA.
(TIF)

S3 Fig Activation of ISGs by KDM5 inhibition is dependent on the cGAS-STING-TBK1-IRF3 pathway. (A) T7 endonuclease assays showing the genome editing efficiency of MCF7 cells with CRISPR/Cas9-mediated TLR3 knockout. (B, C) RT-qPCR (panel B) and western blot (panel C) analyses of control or IRF3 knockout MCF7 cells generated by 3 independent sgRNAs against IRF3 (IRF3-1, IRF3-2, and IRF3-3) after treatment with DMSO or 1 μM KDM5-C70 for 6 days. sgRNA IRF3-1 was used in Fig 3 and panel D. (D) Western blot analysis of control or IRF3 knockout MCF7 cells 5 days after transfection with the indicated siRNAs. (E) RT-qPCR analysis of MCF7 cells with knockout of the indicated genes after treatment with DMSO or 1 μM KDM5-C70 for 6 days. (F) Heatmap showing RT-qPCR analysis of ISGs in MCF7 cells treated with 1 μM KDM5-C70 for 6 days or the indicated concentrations of IFNβ for 24 hours. (G) Illustration of the experimental procedures (left panel) and RT-qPCR analysis of conditioned media treated MCF7 cells (middle and right panels). MCF7 cells with knockout of the indicated genes were pretreated with DMSO or 1 μM KDM5-C70 for 3 days. After washing with PBS 3 times, cells were refed with fresh media without inhibitor and cultured for 2 more days. The media were then collected to treat MCF7 cells for 3 days. (H) Western blot analysis of MCF7 cells with knockout of the indicated genes after treatment with DMSO or 1 μM KDM5-C70 for 6 days. Representative data from triplicate experiments are shown in panel B, E, and G. Error bar denotes SEM. ^#^*p* < 0.01 for inhibitors versus DMSO (panel B and E); KDM5-C70 medium versus mock medium (panel G). ^*p* < 0.01 for knockout sgRNA versus control sgRNA (panel E). The numerical values used to generate graphs in panel B and E–G are available in S1 Data. cGAS, cGAMP synthase; CRISPR/Cas9, clustered regular interspaced short palindromic repeats/CRISPR-associated protein 9; IFN, interferon; IRF3, interferon regulatory factor 3; ISG, interferon-stimulated gene; long, long exposure; RT-qPCR, reverse transcription followed by quantitative PCR; short, short exposure; sgRNA, single guide RNA; siRNA, small interfering RNA; STING, stimulator of interferon genes; TBK1, TANK-binding gene 1; TLR3, toll-like receptor 3.
(TIF)

S4 Fig Induced resistance to virus infection by KDM5 inhibition is dependent on the cGAS-STING-TBK1-IRF3 pathway. (A) Flow cytometry plots (left panel) and quantification of GFP-positive cells (right panel) in MCF7 cells with knockout of the indicated genes 24 hours after infection with VSV-GFP at MOI 0.5. Cells were pretreated with DMSO or 1 μM KDM5-C70 for 5 days, followed by no treatment for 1 day before viral infection. (B) Representative images (left panel) and quantification of relative intensity (right panel) of control or IRF3 knockout MCF7 cells 3 days after infection with vaccinia viruses at MOI 0.25. MCF7 cells were pretreated with DMSO or 1 μM KDM5-C70 for 5 days, followed by no treatment for 1 day before viral infection. (C) qPCR analysis of DNA copy number of vaccinia viruses in growth media from the cells in panel B. Representative data from triplicate experiments are shown in panel C. Three biological replicates are shown in panel A and B. Error bar denotes SEM. ^#^*p* < 0.01 for inhibitors versus DMSO (panel B and C). The numerical values used to generate graphs in panel A–C are available in S1 Data. cGAS, cGAMP synthase; IRF3, interferon regulatory factor 3; MOI, multiplicity of infection; qPCR, quantitative PCR; STING, stimulator of interferon genes; TBK1, TANK-binding kinase 1; VSV-GFP, vesicular stomatitis virus carrying a green fluorescent protein reporter.
(TIF)

S5 Fig KDM5 represses interferon response by inhibiting *STING* expression. (A–D) Western blot analysis of the indicated cell lines after treatment with DMSO or 1 μM KDM5-C70 for 6 days. (E, F) RT-qPCR (panel E) and western blot (panel F) analyses of control or KDM5B/KDM5C double KO MCF7 cells. (G) RT-qPCR analysis of MCF7 cells treated with control or KDM5B/KDM5C siRNAs. (H) Western blot analysis of control or IRF3 KO MCF7 cells 5 days after transfection with the indicated siRNAs. Representative data from triplicate experiments are shown. Error bar denotes SEM. The numerical values used to generate graphs in panel E and G are available in S1 Data. IRF3, interferon regulatory factor 3; KO, knockout; RT-qPCR, reverse transcription followed by quantitative PCR; siRNA, small interfering RNA; STING, stimulator of interferon genes.
(TIF)

S6 Fig KDM5B and KDM5C bind to the promoter of *STING*. (A, B) H3K4me3 ChIP-qPCR analysis of MCF7 cells (panel A) or BT474 cells (panel B) treated with DMSO or 1 μM KDM5-C70 for 1 day. (C) Analysis of ChIP-seq data for KDM5B binding at the *STING* genomic region in K562 cells (GSE29611, upper panel) and KDM5C in ZR-75-30 cells (GSE71327, lower panel) [42]. Heat map showing KDM5B or KDM5C binding on *STING*, but not *cGAS* and downstream genes *OAS2*, *IFI44L*, and *IFI44*. ^#^*p* < 0.01 for the comparisons shown in panel A and B inhibitors versus DMSO. The numerical values used to generate graphs in panel A and B are available in S1 Data. ChIP-seq, chromatin immunoprecipitation; qPCR, quantitative PCR; STING, stimulator of interferon genes.
(TIF)

S7 Fig KDM5-C70 does not affect cytosolic DNA in MCF7 cells, and components of the PRR pathway are efficiently deleted in SKBR3 cells. (A, B) RT-qPCR analysis of MCF7 cells with the indicated treatment. MCF7 cells were treated with 10 μM VE821 for 3 days (panel A) or 1 μM KDM5-C70 for 4 days (panel B), followed by 1-day treatment with 0.2 μM LMB. (C) dsDNA and DAPI staining of MCF7 cells treated with DMSO or 1 μM KDM5-C70 for 3 days. Surface plots of Z-stack images generated with Huygens. Scale bar, 10 μm. (D) Western blot analysis of SKBR3 cells with knockout of the indicated genes. The numerical values used to generate graphs in panel A and B are available in S1 Data. dsDNA, double-stranded DNA; LMB, leptomycin B; PRR, pattern recognition receptor; RT-qPCR, reverse transcription followed by quantitative PCR.
(TIF)

S8 Fig KDM5B is negatively associated with CD8^+^ T-cell infiltration and clinical outcome in cervical cancer. (A) Correlation between KDM5B and STING in TCGA CESC. *n* = 302. (B) Correlation between KDM5B and CXCL10 or STING and CXCL10 in TCGA CESC. (C) Correlation between KDM5B expression and CD8^+^ T-cell infiltration in TCGA CESC. CESC, Cervical Squamous Cell Carcinoma and Endocervical Adenocarcinoma; STING, stimulator of interferon genes; TCGA, The Cancer Genome Atlas.
(TIF)

S1 Table Summary of X-ray data. (DOCX)

S2 Table List of sgRNA targeting sequences. sgRNA, single guide RNA.
(DOCX)

S3 Table List of primers used for RT-qPCR. RT-qPCR, reverse transcription followed by quantitative PCR.
(DOCX)

S4 Table List of primers used for ChIP-qPCR. ChIP-qPCR, chromatin immunoprecipitation followed by quantitative PCR.
(DOCX)

S1 Data Numerical data used in all the figures. (XLSX)

S2 Data List of genes with log_2_FC ≥1 or ≤−1 and *p* < 0.05 in MCF7 cells after treatment with KDM5-C70 for 6 days. (XLSX)

S3 Data List of genes with log_2_FC ≥1 or ≤−1 and *p* < 0.05 in MCF7 cells after treatment with CPI-48 for 6 days. (XLSX)

## Abbreviations

ATM: Ataxia-telangiectasia mutated
ATR: Ataxia-telangiectasia and Rad3-related protein
cGAMP: cyclic GMP-AMP
cGAS: cGAMP synthase
ChIP: chromatin immunoprecipitation
ChIP-seq: ChIP sequencing
CRISPR/Cas9: clustered regular interspaced short palindromic repeats/CRISPR-associated protein 9
CTLA-4: cytotoxic T-cell lymphocyte-associated protein 4
ddC: dideoxycytidine
DNMT: DNA methyltransferase
dsDNA: double-stranded DNA
EZH2: Enhancer of zeste homolog 2
FDR: false discovery rate
GSEA: gene set enrichment analysis
HDAC: histone deacetylase
HPV: human papilloma virus
IC_50_: half maximal inhibitory concentration
IRF: interferon regulatory factor
ISG: interferon-stimulated gene
ISGF3: interferon-stimulated gene factor 3
ISRE: interferon-stimulated response element
LMB: leptomycin
MAVS: mitochondrial antiviral signaling protein
MDA5: melanoma differentiation-associated gene 5
miRNA: microRNA
MOI: multiplicity of infection
mtDNA: mitochondrial DNA
NCBI: National Center for Biotechnology Information
NES: normalized enrichment score
PD-1: programmed cell death protein 1
PRR: Pattern recognition receptor
qPCR: quantitative PCR
RIG-I: retinoic acid inducible gene I
RNA-seq: RNA sequencing
RT-qPCR: reverse transcription followed by quantitative PCR
sgRNA: single guide RNA
siRNA: small interfering RNA
STAT1: signal transducer and activator of transcription 1
STING: stimulator of interferon genes
SUMO: small ubiquitin-like modifier protein
TBK1: TANK-binding kinase 1
TCEP: tris (2-carboxyethyl)phosphine
TCGA: The Cancer Genome Atlas
TLR: toll-like receptor
VSV-GFP: vesicular stomatitis virus carrying a green fluorescent protein reporter
