# Supplementary figures and images for: KDM5 histone demethylases repress immune response via suppression of STING
Source: PLoS Biol. 2018 Aug 6;16(8):e2006134. doi: 10.1371/journal.pbio.2006134 (PMC6095604; doi:10.1371/journal.pbio.2006134)

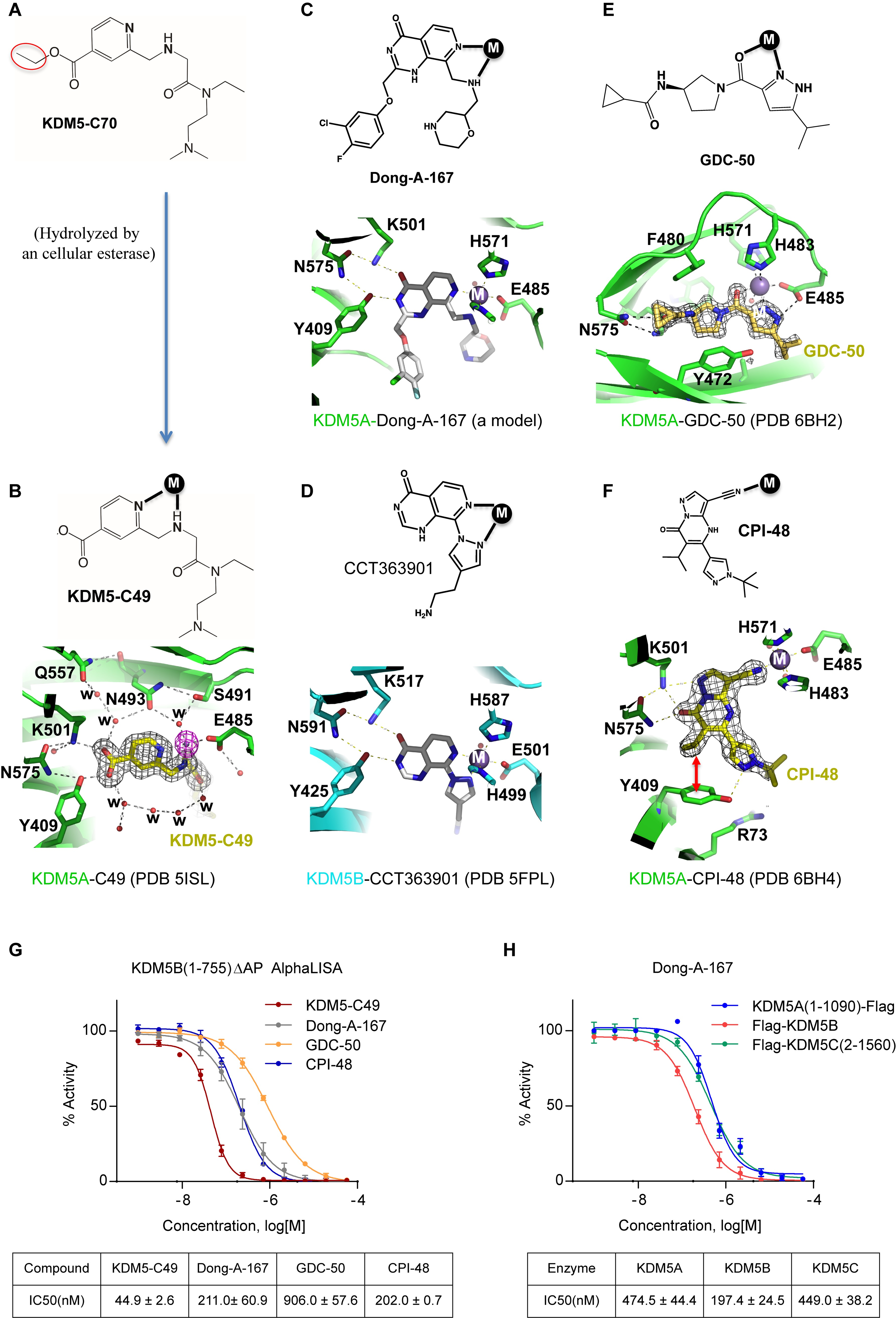

Supplement: S1 Fig — (A) KDM5-C70 was designed as a cell-permeable prodrug that is hydrolyzed by intracellular esterase(s) to generate KDM5-C49, which contains an isonicotinic acid moiety with a carboxylic acid (PCT WO 2014053491) [30, 32]. (B) KDM5-C49 binds in the active site pocket bridging between the metal binding site (magenta) and the hydrogen-bonding network mediated by K501-N575-Y409 (PDB 5ISL) [30]. (C, D) A computer model of Dong-A-167 compound bound in the active site of KDM5A (panel C), based on KDM5B in complex with a related compound containing a pyrido[3,4-d]pyrimidin-4(1H)-one moiety (PDB 5FPL) (D) [86]. (E) GDC-50 (also known as Compound N54) bound in the active site of KDM5A (PDB 6BH2) [36]. (F) Compound CPI-48 bound in the active site of KDM5A. Like GDC-50, Y409 undergoes a conformational change upon the binding of inhibitor, resulting in a van der Waals contact with the isopropyl moiety of CPI-48 (indicated by a red arrow). Crystallographic data of KDM5A-CPI-48 complex in the presence of Mn(II) are shown in S1 Table. (G) AlphaLISA assays showing that KDM5-C70, Dong-A-167, GDC-50, and CPI-48 are potent inhibitors against truncated KDM5B. Shown were representative dose response curves (upper panel) and IC50 values (mean +/− SD) (lower panel) of all 4 compounds against KDM5B (1–755) ΔAP from 3 independent experiments. (H) AlphaLISA assays showing that Dong-A-167 is a potent inhibitor of KDM5A, KDM5B, and KDM5C. Shown were representative dose response curves (upper panel) and IC50 values (mean +/− SD) (lower panel) of Dong-A-167 against KDM5 demethylases from 3 independent experiments. The numerical values used to generate graphs in panel G and H are available in S1 Data. IC50, half maximal inhibitory concentration. (TIF) [file pbio.2006134.s001.tif]

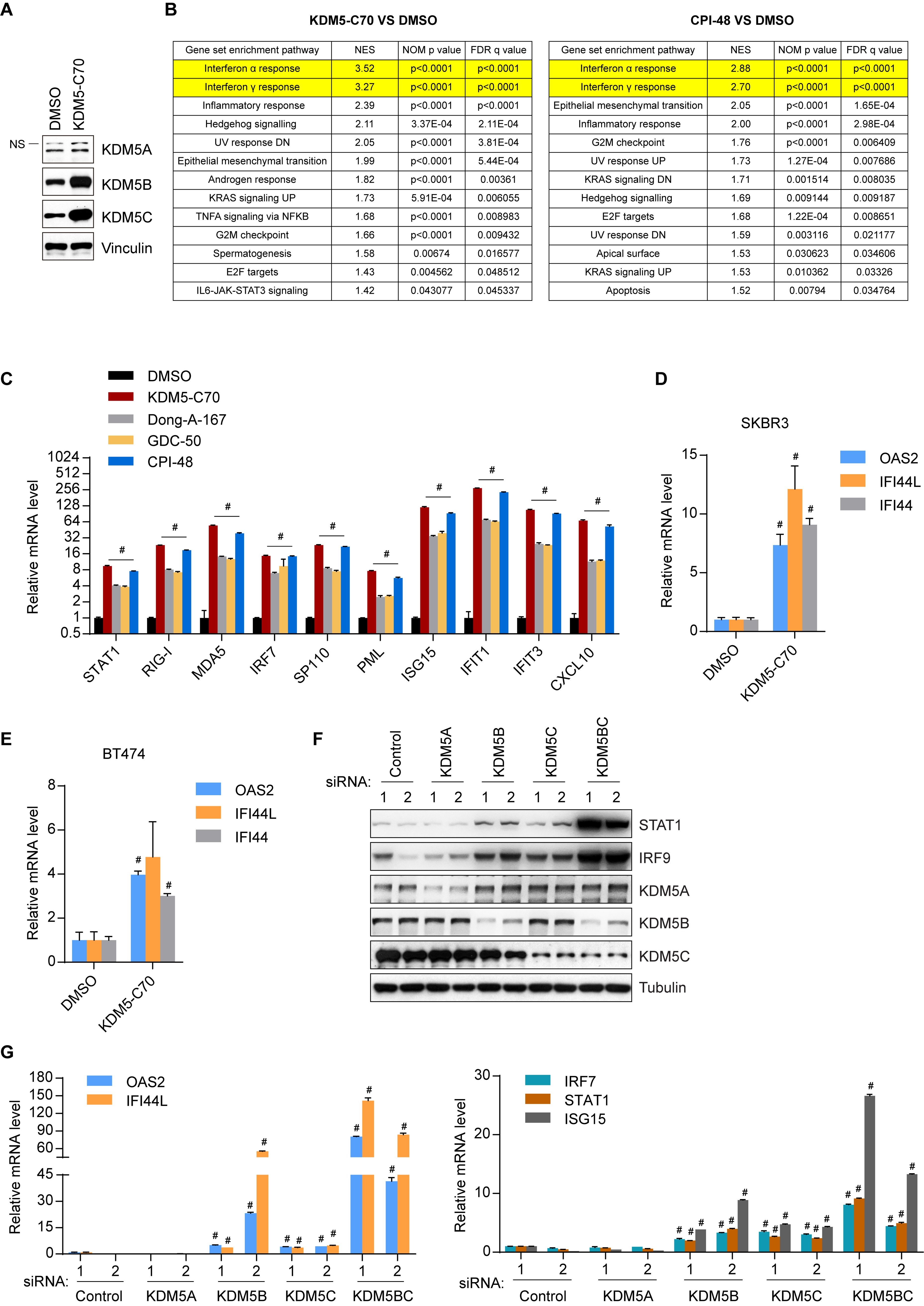

Supplement: S2 Fig — (A) Western blot analyses of MCF7 cells treated with 1 μM KDM5-C70 for 3 days. (B) Pathways that were up-regulated by 3 μM KDM5-C70 or CPI-48 treatment, revealed by GSEA. The gene set database of h. all. v6.1. symbols. gmt (Hallmarks) was used. All the up-regulated pathways in inhibitor-treated cells with FDR q value < 0.05 were shown. (C) RT-qPCR analysis of MCF7 cells treated with 1 μM KDM5-C70, 10 μM each of Dong-A-167, GDC-50, or CPI-48 for 6 days. (D, E) RT-qPCR analysis of SKBR3 cells treated with 5 μM KDM5-C70 (panel D) and BT474 cells treated with 1 μM KDM5-C70 (panel E) for 6 days. (F, G) Western blot (panel F) and RT-qPCR (panel G) analyses of MCF7 cells 5 days after transfection with the indicated siRNAs. KDM5BC, 2 siRNAs targeting KDM5B and KDM5C. Representative data from triplicate experiments are shown. Error bar denotes SEM. #p < 0.01 for inhibitors versus DMSO (panel C–E), for KDM5 siRNA versus average of 2 control siRNAs (panel G). The numerical values used to generate graphs in panel C–E and G are available in S1 Data. Control, universal negative control; FDR q value, false discovery rate q value; GSEA, gene set enrichment analysis; NES, normalized enrichment score; NS, nonspecific band; NOM p value, nominal p-value; RT-qPCR, RT-qPCR, reverse transcription followed by quantitative PCR; siRNA, small interfering RNA. (TIF) [file pbio.2006134.s002.tif]

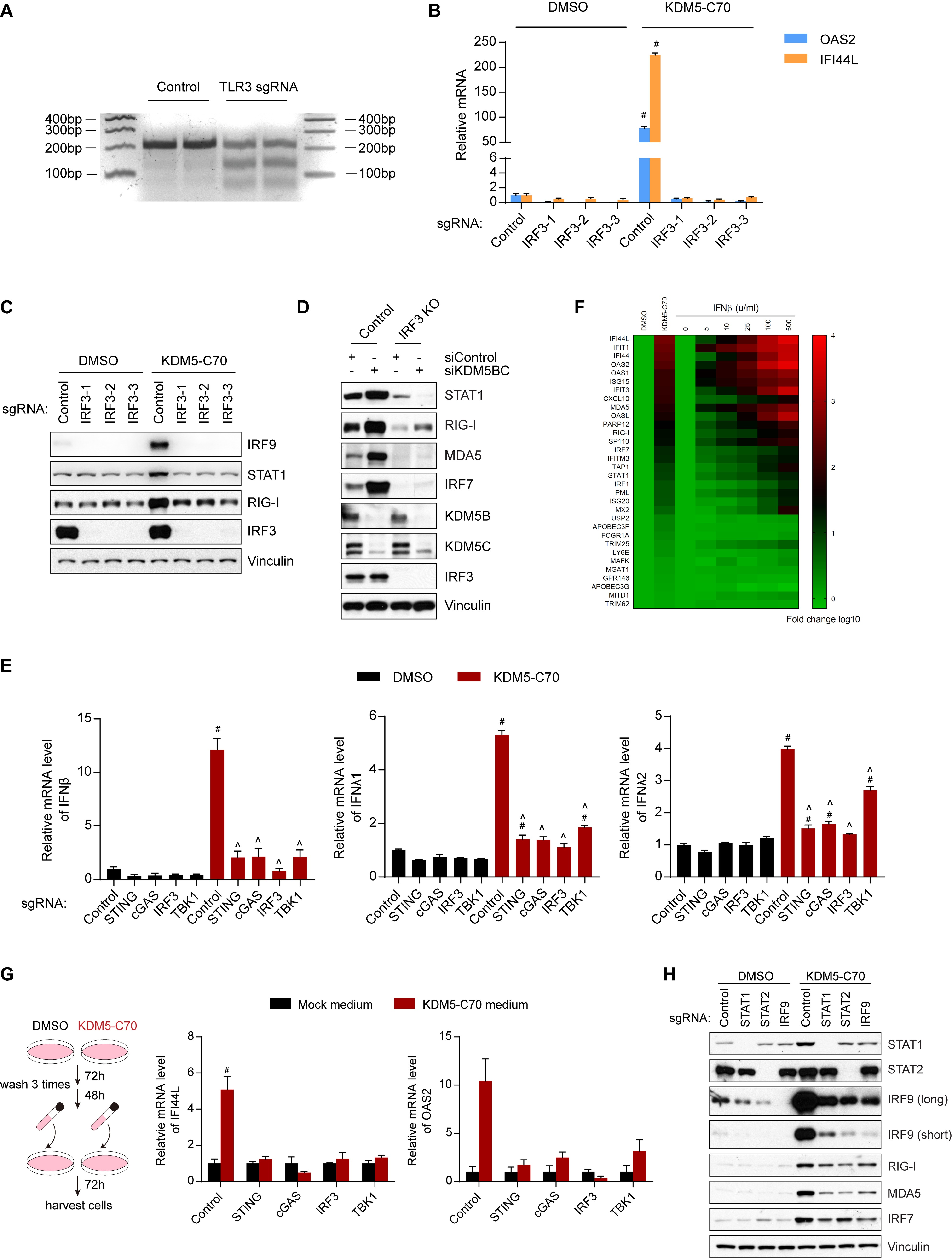

Supplement: S3 Fig — (A) T7 endonuclease assays showing the genome editing efficiency of MCF7 cells with CRISPR/Cas9-mediated TLR3 knockout. (B, C) RT-qPCR (panel B) and western blot (panel C) analyses of control or IRF3 knockout MCF7 cells generated by 3 independent sgRNAs against IRF3 (IRF3-1, IRF3-2, and IRF3-3) after treatment with DMSO or 1 μM KDM5-C70 for 6 days. sgRNA IRF3-1 was used in Fig 3 and panel D. (D) Western blot analysis of control or IRF3 knockout MCF7 cells 5 days after transfection with the indicated siRNAs. (E) RT-qPCR analysis of MCF7 cells with knockout of the indicated genes after treatment with DMSO or 1 μM KDM5-C70 for 6 days. (F) Heatmap showing RT-qPCR analysis of ISGs in MCF7 cells treated with 1 μM KDM5-C70 for 6 days or the indicated concentrations of IFNβ for 24 hours. (G) Illustration of the experimental procedures (left panel) and RT-qPCR analysis of conditioned media treated MCF7 cells (middle and right panels). MCF7 cells with knockout of the indicated genes were pretreated with DMSO or 1 μM KDM5-C70 for 3 days. After washing with PBS 3 times, cells were refed with fresh media without inhibitor and cultured for 2 more days. The media were then collected to treat MCF7 cells for 3 days. (H) Western blot analysis of MCF7 cells with knockout of the indicated genes after treatment with DMSO or 1 μM KDM5-C70 for 6 days. Representative data from triplicate experiments are shown in panel B, E, and G. Error bar denotes SEM. #p < 0.01 for inhibitors versus DMSO (panel B and E); KDM5-C70 medium versus mock medium (panel G). ^p < 0.01 for knockout sgRNA versus control sgRNA (panel E). The numerical values used to generate graphs in panel B and E–G are available in S1 Data. cGAS, cGAMP synthase; CRISPR/Cas9, clustered regular interspaced short palindromic repeats/CRISPR-associated protein 9; IFN, interferon; IRF3, interferon regulatory factor 3; ISG, interferon-stimulated gene; long, long exposure; RT-qPCR, reverse transcription followed by quantitative PCR; short [file pbio.2006134.s003.tif]

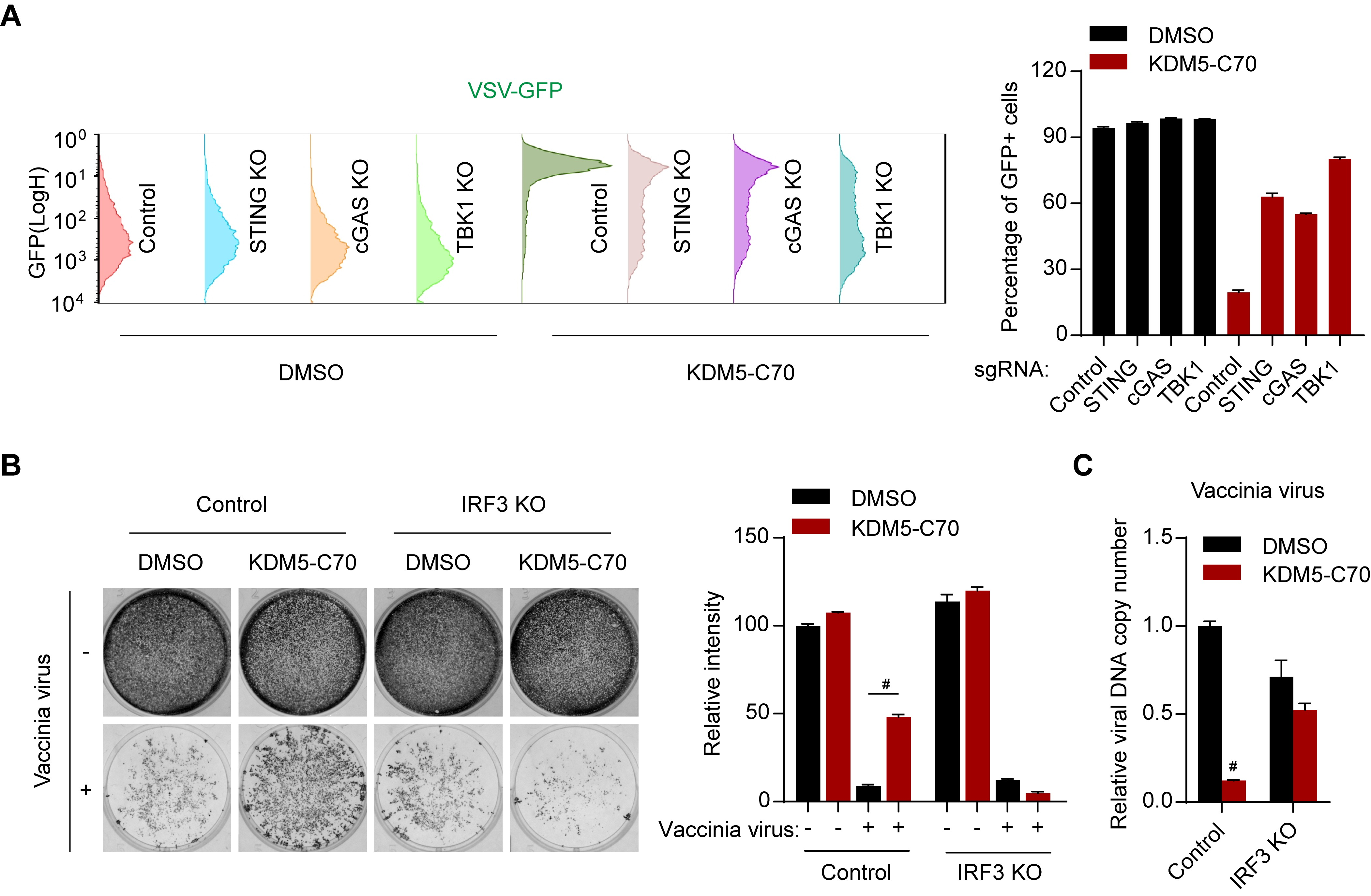

Supplement: S4 Fig — (A) Flow cytometry plots (left panel) and quantification of GFP-positive cells (right panel) in MCF7 cells with knockout of the indicated genes 24 hours after infection with VSV-GFP at MOI 0.5. Cells were pretreated with DMSO or 1 μM KDM5-C70 for 5 days, followed by no treatment for 1 day before viral infection. (B) Representative images (left panel) and quantification of relative intensity (right panel) of control or IRF3 knockout MCF7 cells 3 days after infection with vaccinia viruses at MOI 0.25. MCF7 cells were pretreated with DMSO or 1 μM KDM5-C70 for 5 days, followed by no treatment for 1 day before viral infection. (C) qPCR analysis of DNA copy number of vaccinia viruses in growth media from the cells in panel B. Representative data from triplicate experiments are shown in panel C. Three biological replicates are shown in panel A and B. Error bar denotes SEM. #p < 0.01 for inhibitors versus DMSO (panel B and C). The numerical values used to generate graphs in panel A–C are available in S1 Data. cGAS, cGAMP synthase; IRF3, interferon regulatory factor 3; MOI, multiplicity of infection; qPCR, quantitative PCR; STING, stimulator of interferon genes; TBK1, TANK-binding kinase 1; VSV-GFP, vesicular stomatitis virus carrying a green fluorescent protein reporter. (TIF) [file pbio.2006134.s004.tif]

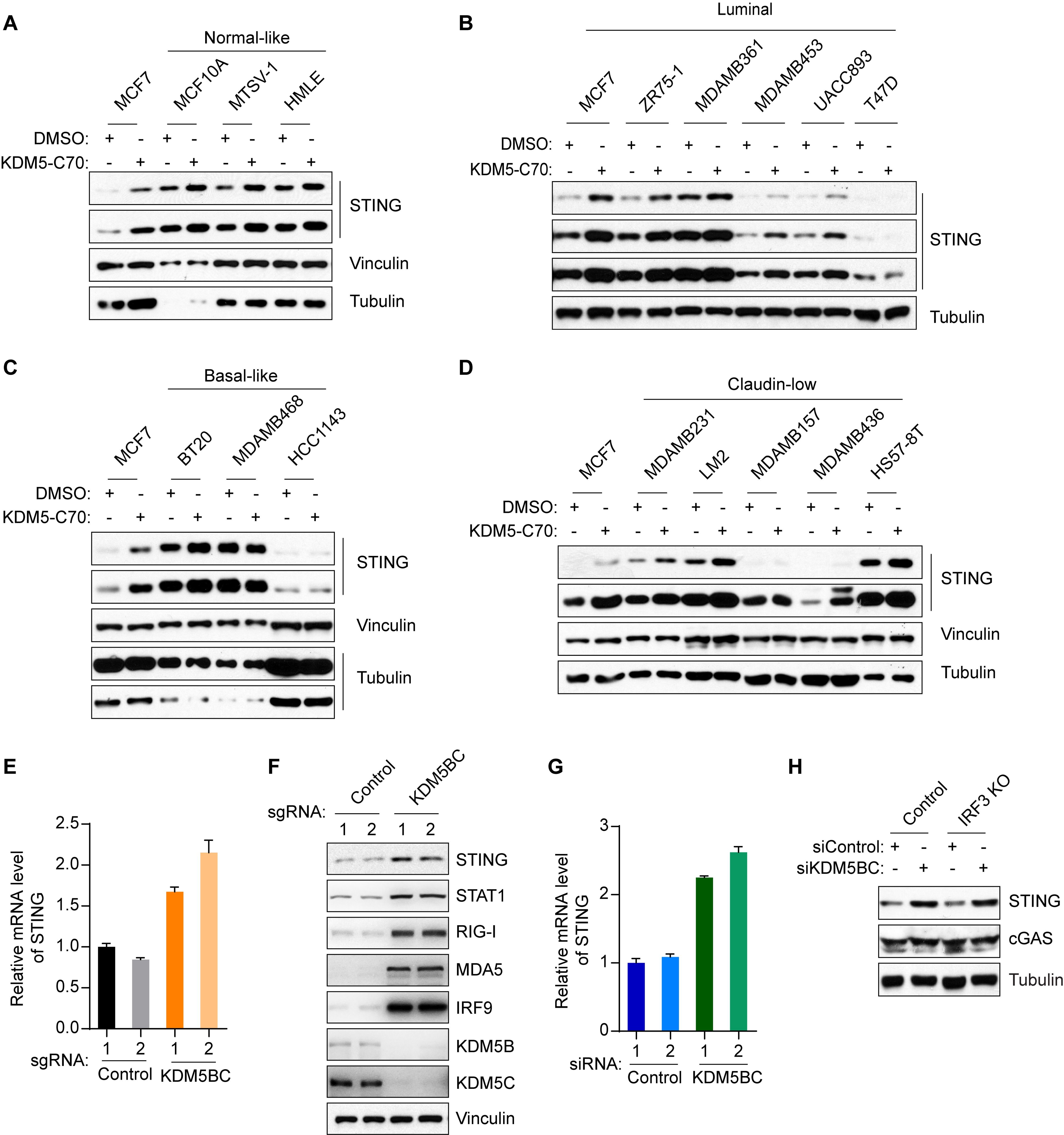

Supplement: S5 Fig — (A–D) Western blot analysis of the indicated cell lines after treatment with DMSO or 1 μM KDM5-C70 for 6 days. (E, F) RT-qPCR (panel E) and western blot (panel F) analyses of control or KDM5B/KDM5C double KO MCF7 cells. (G) RT-qPCR analysis of MCF7 cells treated with control or KDM5B/KDM5C siRNAs. (H) Western blot analysis of control or IRF3 KO MCF7 cells 5 days after transfection with the indicated siRNAs. Representative data from triplicate experiments are shown. Error bar denotes SEM. The numerical values used to generate graphs in panel E and G are available in S1 Data. IRF3, interferon regulatory factor 3; KO, knockout; RT-qPCR, reverse transcription followed by quantitative PCR; siRNA, small interfering RNA; STING, stimulator of interferon genes. (TIF) [file pbio.2006134.s005.tif]

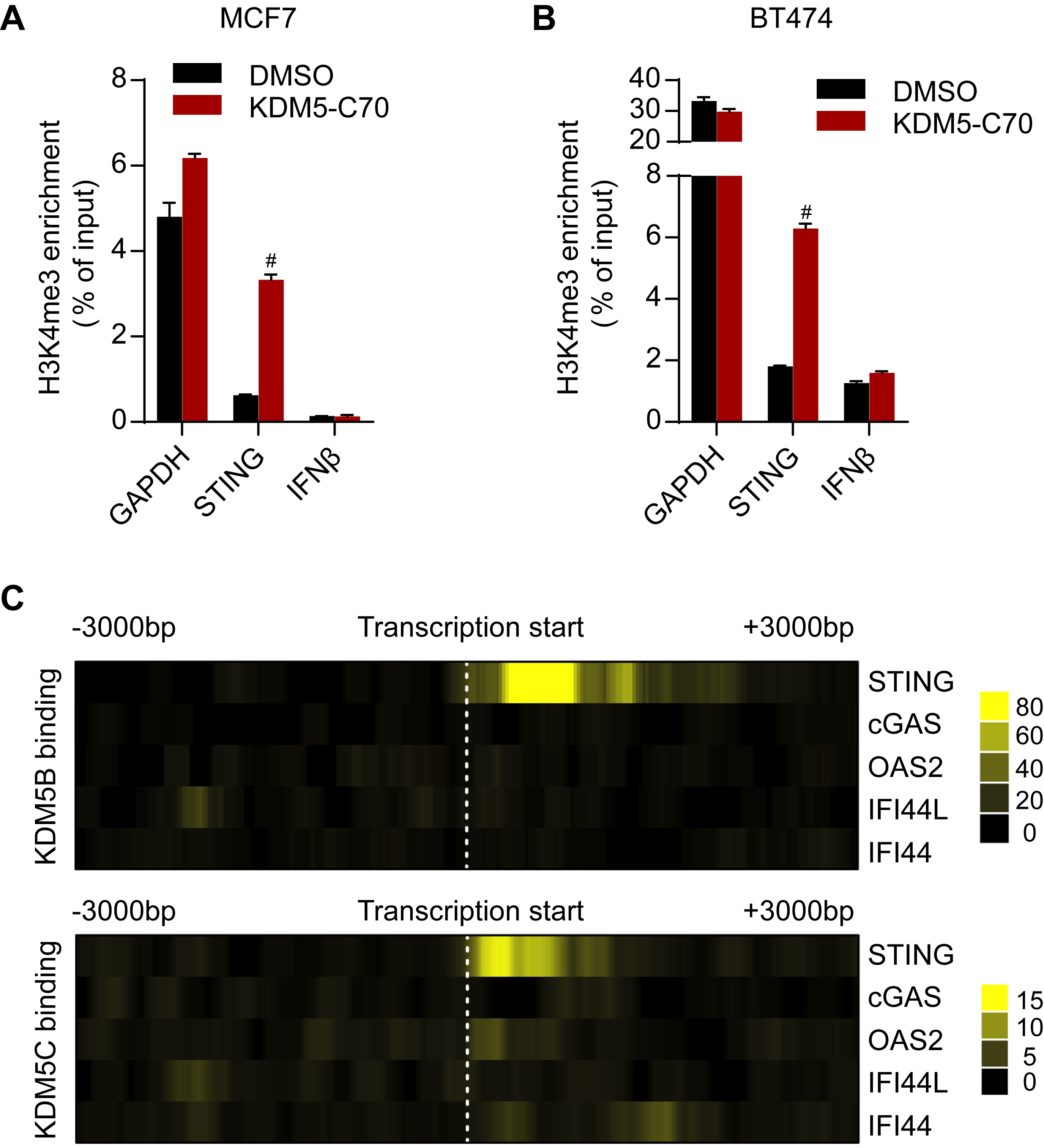

Supplement: S6 Fig — (A, B) H3K4me3 ChIP-qPCR analysis of MCF7 cells (panel A) or BT474 cells (panel B) treated with DMSO or 1 μM KDM5-C70 for 1 day. (C) Analysis of ChIP-seq data for KDM5B binding at the STING genomic region in K562 cells (GSE29611, upper panel) and KDM5C in ZR-75-30 cells (GSE71327, lower panel) [42]. Heat map showing KDM5B or KDM5C binding on STING, but not cGAS and downstream genes OAS2, IFI44L, and IFI44. #p < 0.01 for the comparisons shown in panel A and B inhibitors versus DMSO. The numerical values used to generate graphs in panel A and B are available in S1 Data. ChIP-seq, chromatin immunoprecipitation; qPCR, quantitative PCR; STING, stimulator of interferon genes. (TIF) [file pbio.2006134.s006.tif]

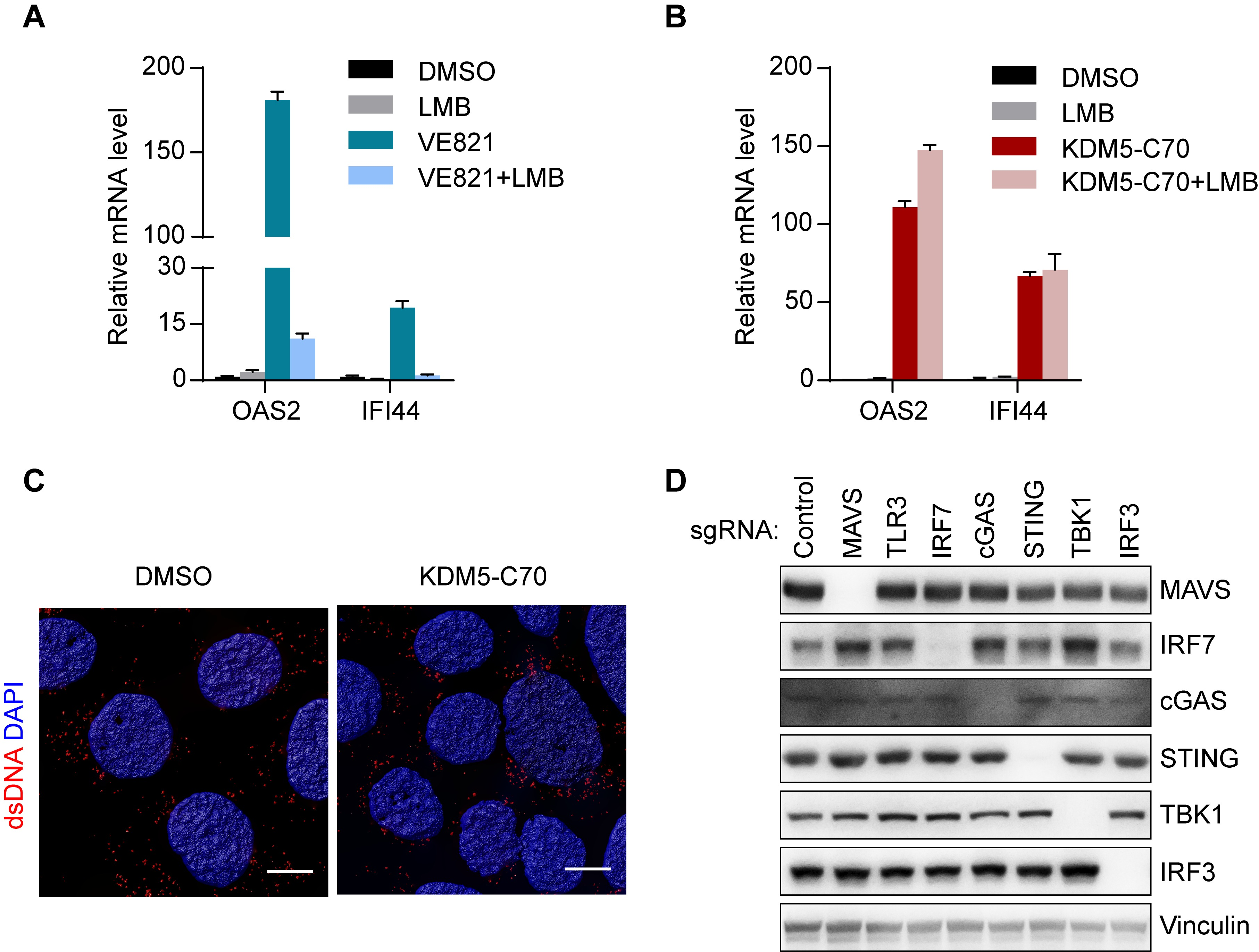

Supplement: S7 Fig — (A, B) RT-qPCR analysis of MCF7 cells with the indicated treatment. MCF7 cells were treated with 10 μM VE821 for 3 days (panel A) or 1 μM KDM5-C70 for 4 days (panel B), followed by 1-day treatment with 0.2 μM LMB. (C) dsDNA and DAPI staining of MCF7 cells treated with DMSO or 1 μM KDM5-C70 for 3 days. Surface plots of Z-stack images generated with Huygens. Scale bar, 10 μm. (D) Western blot analysis of SKBR3 cells with knockout of the indicated genes. The numerical values used to generate graphs in panel A and B are available in S1 Data. dsDNA, double-stranded DNA; LMB, leptomycin B; PRR, pattern recognition receptor; RT-qPCR, reverse transcription followed by quantitative PCR. (TIF) [file pbio.2006134.s007.tif]

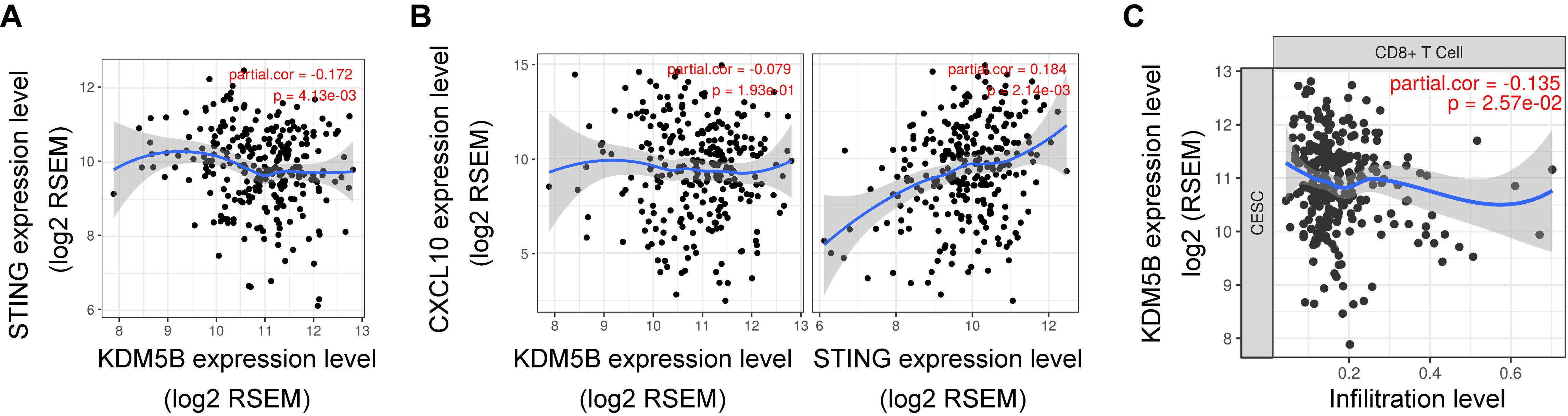

Supplement: S8 Fig — (A) Correlation between KDM5B and STING in TCGA CESC. n = 302. (B) Correlation between KDM5B and CXCL10 or STING and CXCL10 in TCGA CESC. (C) Correlation between KDM5B expression and CD8+ T-cell infiltration in TCGA CESC. CESC, Cervical Squamous Cell Carcinoma and Endocervical Adenocarcinoma; STING, stimulator of interferon genes; TCGA, The Cancer Genome Atlas. (TIF) [file pbio.2006134.s008.tif]
