# Supplementary material for: KDM5 histone demethylases repress immune response via suppression of STING
Source: PLoS Biol. 2018 Aug 6;16(8):e2006134. doi: 10.1371/journal.pbio.2006134 (PMC6095604; doi:10.1371/journal.pbio.2006134)
Supplement: S1 Table — (DOCX) [file pbio.2006134.s009.docx]

**S1 Table. Summary of X-ray data.**
