# Supplementary material for: KDM5 histone demethylases repress immune response via suppression of STING
Source: PLoS Biol. 2018 Aug 6;16(8):e2006134. doi: 10.1371/journal.pbio.2006134 (PMC6095604; doi:10.1371/journal.pbio.2006134)
Supplement: S2 Table — sgRNA, single guide RNA. (DOCX) [file pbio.2006134.s010.docx]

**S2 Table. List of sgRNA targeting sequences.**

| KDM5A-1 | CTGCAAAATTCGGCCGCCCA |
| --- | --- |
| KDM5A-2 | CGTCTTTGAGCCGAGTTGGG |
| KDM5B-1 | GCGCGGGCCTGGGTGCAGTG |
| KDM5B-2 | GGAGGCAGGAACTCGCCCAG |
| KDM5C-1 | GTCGGACCCCGGCTCCATGG |
| KDM5C-2 | AGATTCCCAATGTAGAACGG |
| RIG-I | AGGTTGTTCACAAGAATCTG |
| MDA-5 | TGAAGCACGAGATGAGATAG |
| MAVS | GGGTATTGAAGAGATGCCAG |
| IRF7 | AGGGCACGCGGAAACAGGTG |
| cGAS | CGAGGCCGCCCTGCCTAAGG |
| STING | TGAGTCACCTGGAGTGGATG |
| TBK1 | AGAGCACTTCTAATCATCTG |
| IRF3-1 | GGGCAGGATCCGTGGCTTTG |
| IRF3-2 | TGGTGTCGCAGCTGGACCTG |
| IRF3-3 | GGCCGTGCTTCCAAGGGATG |
| TLR3 | TGCACGGGCTTTTCAATGTG |
| STAT1 | GAGGTCATGAAAACGGATGG |
| STAT2 | GGTGCAGCTGATCCTGAAAG |
| IRF9 | GCTGGCCTGTGGAATTGTTG |
