# Supplementary material for: KDM5 histone demethylases repress immune response via suppression of STING
Source: PLoS Biol. 2018 Aug 6;16(8):e2006134. doi: 10.1371/journal.pbio.2006134 (PMC6095604; doi:10.1371/journal.pbio.2006134)
Supplement: S3 Table — RT-qPCR, reverse transcription followed by quantitative PCR. (DOCX) [file pbio.2006134.s011.docx]

| **S3 Table. List of primers used for RT-qPCR.** | |
| --- | --- |
| GAPDH-F | TGCACCACCAACTGCTTAGC |
| GAPDH-R | GGCATGGACTGTGGTCATGAG |
| OAS2-F | CAGTCCTGGTGAGTTTGCAGT |
| OAS2-R | ACAGCGAGGGTAAATCCTTGA |
| IFI44L-F | GAGCACAGAAATAGGCTTCTAGC |
| IFI44L-R | TGGTATCAGACCCCACTACGG |
| IRF7-F | GAGCCGTACCTGTCACCCT |
| IRF7-R | GGGCCGTATAGGAACGTGC |
| SP110-F | CTGCTGAAGCGGAAAAACTCG |
| SP110-R | CAGGGTCTTAGATACATGATGGC |
| PML-F | CAGCTCGGAAGACTCAGATGC |
| PML-R | CTGGAGGTCACTGGACTCACT |
| STAT1-F | AGGAAAAGCAAGCGTAATCTTCA |
| STAT1-R | TATTCCCCGACTGAGCCTGAT |
| IFIT1-F | CTGAATGCAGCTCACCTCTG |
| IFIT1-R | GGATGGAATTGCCTGCTAGA |
| IFIT3-F | CTGAACTGCTCAGCCCACA |
| IFIT3-R | TCAGCTTGCCCTAAGCACTC |
| STING-F | GAGGAGGAGGAGGCTGAGTT |
| STING-R | ACCGCAAGTGAGAGGGAGTA |
| IRF1-F | AGCTCAGCTGTGCGAGTGTA |
| IRF1-R | TAGCTGCTGTGGTCATCAGG |
| ISG20-F | CTTCCAGGCACTGAAAGAGG |
| ISG20-R | ATCTTCCACCGAGCTGTGTC |
| OAS1-F | CAAGCTCAAGAGCCTCATCC |
| OAS1-R | TGGGCTGTGTTGAAATGTGT |
| IFITM3-F | TCGCCTACTCCGTGAAGTCT |
| IFITM3-R | CATAGGCCTGGAAGATCAGC |
| APOBEC3G-F | GTTCAGCAAGTGGAGGAAGC |
| APOBEC3G-R | AGTAGTAGAGGCGGGCAACA |
| MGAT1-F | GGTGGAGAAAGTGAGGACCA |
| MGAT1-R | CGGAACTGGAAGGTGACAAT |
| APOBEC3F-F | GAATACCGTCTGGCTGTGCT |
| APOBEC3F-R | AGGGGGTCCAGGATACAAAC |
| GPR146-F | CTTTGTCAACATGGCAGTGG |
| GPR146-R | AGTGCACGCTCGATGTAGTG |
| LY6E-F | TGATGTGCTTCTCCTGCTTG |
| LY6E-R | ACAGGTCTTGCTCAGGCTGT |
| FCGR1A-F | GCCCTCTCTTTTGAGGCTCT |
| FCGR1A-R | CTATGGTATCGCCGCTTCTC |
| TRIM62-F | ACTACTGGGAGGTGGTGGTG |
| TRIM62-R | CAGGAAGACACCCACCTTGT |
| USP2-F | CTTCTGGGACCTCTCACTGC |
| USP2-R | TGTTGTGAGCTTGCTGGTTC |
| MAFK-F | GCAGAAGGAGGAGCTGGAG |
| MAFK-R | GAGAGCTCGGTGGACTTGAC |
| PARP12-F | GCAGTGCATCAAGCTCCATA |
| PARP12-R | CCTGTGGGACAAAAAGAGGA |
| TRIM25-F | GATGTGAGAAACAGGCAGCA |
| TRIM25-R | ATCCCTCTTGGTCAGGCTCT |
| MITD1-F | CTAGATTCGGAGTCGCGGTA |
| MITD1-R | GTTTTCCGCTCTGTCCATGT |
| MX2-F | AAGCAGTATCGAGGCAAGGA |
| MX2-R | TCGTGCTCTGAACAGTTTGG |
| OASL-F | CCACTTGACAGTGGAGCAGA |
| OASL-R | GGGATGGTCTCCAGCAGATA |
| TAP1-F | ACGTCCACCCTGAGTGATTC |
| TAP1-R | TGGACTTTGCCAGAGATTCC |
