# Supplementary material for: KDM5 histone demethylases repress immune response via suppression of STING
Source: PLoS Biol. 2018 Aug 6;16(8):e2006134. doi: 10.1371/journal.pbio.2006134 (PMC6095604; doi:10.1371/journal.pbio.2006134)
Supplement: S4 Table — ChIP-qPCR, chromatin immunoprecipitation followed by quantitative PCR. (DOCX) [file pbio.2006134.s012.docx]

**S4 Table. List of primers used for ChIP-qPCR.**

| gGAPDH-F | TACTAGCGGTTTTACGGGCG |
| --- | --- |
| gGAPDH-R | TCGAACAGGAGGAGCAGAGAGCGA |
| gSTING-F | GGCAATGGAATGGAGGCTTTCTC |
| gSTING-R | TTCTCCACAACACTCTAGCCCTG |
| gSTING downstream (after the last exon)-F | CCTGTCCTTTCAGTGCCTTTCT |
| gSTING downstream (after the last exon)-R | GGCTCTCTCATCTGGCACCTAT |
| gIFNβ-F | ACAGGTAGTAGGCGACACTGTTC |
| gIFNβ-R | CCATTCAATTGCCACAGGAGCTT |
| gNDUFA9-F | GCAGACACCTGAGTGTGCAT |
| gNDUFA9-R | CAAGAACGAGGGGAAAAGTG |
| gOAS2-F | TTTCAGTTTCCTGGCTCTGG |
| gOAS2-R | CCATTTCCCATTGCTCTCAG |
| gIFI44L-F | ACCGTGGCTGCTCGATAAAT |
| gIFI44L-R | TCGCTTACCTGTTTCTAGGG |
